# *In vivo* and *ex vivo* cardiovascular haemodynamic responses of preterm growth-restricted lambs to perinatal asphyxia

**DOI:** 10.1042/CS20258191

**Published:** 2025-12-23

**Authors:** Zahrah Azman, Beth R. Piscopo, Amy E. Sutherland, Alison Thiel, Valerie A. Zahra, Yen Pham, Ilias Nitsos, Mumu Mahjabin Hossain, Atul Malhotra, Suzanne L. Miller, Kristen J. Bubb, Graeme R. Polglase, Beth J. Allison

**Affiliations:** 1 The Ritchie Centre, Hudson Institute of Medical Research, Clayton, VIC, Australia; 2 Department of Obstetrics and Gynaecology, Monash University, Clayton, VIC, Australia; 3 Monash Newborn, Monash Medical Centre, Clayton, VIC, Australia; 4 Department of Paediatrics, Monash University, Clayton, VIC, Australia; 5 Biomedicine Discovery Institute, Monash University, Clayton, VIC, Australia; 6 Victorian Heart Institute, Monash University, Clayton, VIC, Australia

**Keywords:** cardiovascular, fetal growth restriction, perinatal asphyxia, preterm birth

## Abstract

Fetal growth restriction (FGR) arises from chronic hypoxia and increases the risk of cardiovascular dysfunction following perinatal asphyxia, although underlying mechanisms remain unclear. We investigated whether cardiovascular responses to asphyxia are impaired in preterm FGR lambs and whether this arises from α_1_- and β_1_-adrenergic receptor dysfunction. Ewes underwent sterile fetal surgery at 89 days’ gestation (d; term=148 d) to induce FGR (single umbilical artery ligation) or sham surgery (control). At 126 d, lambs were delivered, instrumented and randomised to immediate ventilation (Control_VENT_ *n*=6; FGR_VENT_ *n*=6) or asphyxia (Control_ASPHYXIA_ *n*=12; FGR_ASPHYXIA_ *n*=11) by umbilical cord occlusion until diastolic blood pressure (BP) decreased to 10 mmHg. Lambs were ventilated for 8 hours before baseline ex vivo cardiac function was assessed via Langendorff perfusion to measure left ventricular developed pressure (LVDP), heart rate (HR) and coronary perfusion pressure (CPP). *Ex vivo* α_1_- and β_1_-adrenergic responses were assessed via phenylephrine and dobutamine administration, respectively. FGR_ASPHYXIA_ lambs had lower BP during asphyxia and took longer to reach a diastolic BP of 10 mmHg (*P*<0.05 vs Control_ASPHYXIA_). FGR_ASPHYXIA_ lambs had lower BP in the first 5 minutes after return of spontaneous circulation due to impaired vascular contractility (*P*<0.05 vs ControlASPHYXIA). Baseline LVDP, HR and CPP were similar between groups. FGRASPHYXIA lambs had increased LVDP responses to phenylephrine and dobutamine (*P*<0.05 vs FGR_VENT_ and Control_ASPHYXIA_), without significant changes to HR or CPP. Overall, FGR lambs exhibit impaired vascular contractility and heightened cardiac α_1_- and β_1_-adrenergic responsiveness after perinatal asphyxia, consistent with reduced autonomic regulation, potentially increasing susceptibility to cardiovascular dysfunction postnatally.

## Introduction

Perinatal asphyxia is the largest contributor to neonatal mortality worldwide and describes a period of severe oxygen deprivation or hypoxia in the immediate period surrounding birth [1]. Asphyxiated infants undergo a sequence of cardiovascular changes involving the redistribution of cardiac output to maintain adequate perfusion and oxygenation of critical organs such as the brain, heart and adrenal glands [2]. Increasing exposure to hypoxia leads to cardiovascular dysfunction, ultimately resulting in low cardiac output, reduced myocardial contractility secondary to poor perfusion and ischaemic cardiac injury [2,3]. Importantly, clinical and preclinical data have shown that perinatal asphyxia significantly increases the risk of long-term cardiovascular disease [4,5]. Current resuscitation guidelines are not tailored to personalised care and therefore do not consider the influence of pre-existing perinatal complications, such as fetal growth restriction (FGR) [6].

FGR is a pregnancy complication describing a foetus that fails to reach its biological growth potential following placental insufficiency, resulting in a state of chronic hypoxaemia [7,8]. FGR greatly increases the risk of cardiorespiratory complications in the neonatal period, including respiratory distress [9], systolic and diastolic cardiac dysfunction [10–12], and impaired autonomic control [13]. Many FGR infants are also delivered preterm to prevent stillbirth [14–16], which may further compromise cardiovascular function in the neonatal period [17]. These factors are likely to increase the vulnerability of FGR infants experiencing perinatal asphyxia. Clinical cohort studies have demonstrated no clear consensus on whether there is a greater incidence of asphyxia in FGR infants [9,18–20]. Regardless, FGR infants exposed to a second hit in the form of asphyxia have an increased risk of developing poorer outcomes following asphyxia, such as moderate to severe metabolic acidosis [21]. However, the physiology and mechanisms of the cardiovascular response to an asphyxia-induced acute hypoxia following FGR are poorly characterised.

In the foetus, acute hypoxia triggers the activation of compensatory mechanisms to maintain oxygen delivery to critical organs [22]. Acute hypoxia is detected by chemoreceptors in the carotid body, resulting in the redistribution of cardiac output to vital organs via the brainstem, led by an increased catecholamine production, increased sympathetic activity and peripheral vasoconstriction [22,23]. Chronic hypoxemia, as in FGR, is associated with a sustained redistribution of substrate delivery towards essential organs, mediated by long-term alterations in vascular tone and adrenergic signalling rather than the acute reflex responses of short-term hypoxia [24]. Several preclinical sheep studies have demonstrated that chronic hypoxia alters the cardiovascular responses to a secondary acute hypoxia, both *in utero* and in the immediate perinatal period, highlighting key deficiencies in the systems required to adequately tolerate secondary acute hypoxia [25–28]. Preterm birth is also associated with altered cardiac adrenergic receptor expression [29], which could exacerbate the cardiovascular vulnerabilities associated with FGR. Impairments in both α_1_- and β_1_-adrenergic signalling may therefore underlie the impeded ability of preterm FGR neonates to defend against superimposed challenges; however, these mechanisms remain unexplored in asphyxia.

The responses of preterm FGR lambs to mild perinatal asphyxia (end-diastolic BP decline to 25 mmHg) have been previously characterised, demonstrating an apparent tolerance to asphyxia [27]. However, the mechanisms underlying this response are unknown, and it is unclear whether FGR lambs are able to tolerate a more severe asphyxic insult [end-diastolic blood pressure (BP) decline to 10 mmHg]. In the current study, we aimed to: (i) investigate the ability of preterm FGR lambs to mount an adaptive cardiovascular response to severe perinatal asphyxia and (ii) determine whether altered cardiovascular responses are attributed to altered adrenergic receptor signalling. We hypothesised that FGR lambs would demonstrate a tolerance to severe perinatal asphyxia (end-diastolic BP decline to 10 mmHg) but would experience a compromised cardiovascular response in the recovery period due to a diminished capacity to activate myocardial α_1_- and β_1_-adrenergic receptor (α_1_- and β_1_-AR) signalling.

## Methods

### Ethical approval

Experimental procedures were approved by the Monash Medical Centre Animal Ethics Committee A (approval numbers MMCA 2022/08 and 2022/16). Experimental procedures were conducted in accordance with the National Health and Medical Research Council of Australia Code of Practice for the Care and Use of Animals for Scientific Purposes and ARRIVE (Animal Research: Reporting In Vivo Experiments) Guidelines, version 2.0.

### Animal care and surgical preparation

Singleton or twin-bearing pregnant Border-Leicester ewes (*n*=22) were obtained from the Monash Animal Research Platform (MARP) Gippsland Field Station and acclimatised at the MARP Clayton facility for one week. Ewes were transported to the Monash Health Translation Precinct Animal Facility at least five days prior to surgery, where they were housed for the duration of experimentation in individual floor pens in a temperature-controlled environment (20 ± 1°C; relative humidity 50 ± 10%) under a 12-hour light/dark cycle. Ewes were fed twice daily with a lucerne chaff mixture and had *ad libitum* access to water. Ewes were fasted from food, but not water, for a minimum of 16 hours prior to surgery.

Ewes underwent surgery at 89 days’ gestation (d; term=148 d) to induce early onset FGR. The SUAL model of FGR induces early onset placental insufficiency and asymmetrical FGR consistent with clinical definitions of FGR [30]. Briefly, at 89 ± 1 days’ gestational age (dGA; ~0.6 gestation, term~148 dGA), anaesthesia was induced through intravenous administration of sodium thiopentone via the jugular vein (20 mg/kg pentothal, Boehringer Ingelheim, NSW, Australia). Anaesthesia was maintained via inhaled isoflurane (1–5% in 10/30% O_2_/N_2_O; Bomac Animal Health, NSW, Australia) following intubation with a cuffed endotracheal tube (internal diameter 8.0 mm, outer diameter 10.9 mm; Portex, England) delivering positive pressure ventilation (EV500 Anaesthesia Ventilator, ULCO Medical Engineering, NSW, Australia). Antibiotics (1 g ampicillin sodium, Aspen Pharmacare Australia, and 500 mg oxytetracycline hydrochloride; Engemycin-100, Coopers Animal Health, Australia) were administered intravenously to the ewe and maintained for three days post-surgery. A laparotomy was performed to expose the fetal hindlimbs and umbilical cord before a small incision was made in the umbilical cord sheath. In the case of a twin pregnancy, one foetus was randomly assigned to undergo the SUAL procedure while the other was assigned as the control (sham surgery). SUAL was performed by isolating one of the two umbilical arteries before two silk sutures (2/0 Polysorb braided suture, Covidien, Metronic, Minneapolis, U.S.A.) were tied tightly around the artery. The sham-SUAL (control) twin had its umbilical cord manipulated but not ligated. Each foetus was returned to the uterus, and the abdominal incisions were closed. Anaesthesia was withdrawn, and mechanical ventilation was ceased once the ewe commenced regular spontaneous breathing. The ewe was extubated upon return of a swallowing reflex and returned to her cage, where food and water were provided. Paracetamol was administered to provide additional analgesic coverage, either via a suppository tablet at the completion of surgery (500 mg Panadol, GSK, Australia) or orally (1 g Panadol) for three days post-surgery. Ewes were monitored daily from surgery until experimentation and post-mortem.

30

### Asphyxia and resuscitation

Ewes received intramuscular betamethasone (11.4 mg; Celestone Chronodose, Schering Plough, Australia) at 124 and 125 d to rapidly mature critical organs such as the lung. At 126 d (~0.85 gestation; equivalent to human cardiovascular development at 32–34 weeks’ gestation [31–33]), ewes were anaesthetised (20 mg/kg pentothal and maintained in inhaled isoflurane; 1–5% in 10/30% O_2_/N_2_O), and a caesarean section was performed to partially exteriorise the foetus from the uterus. Lambs were instrumented as previously described [27]. Briefly, the foetus was intubated with an appropriately sized cuffed endotracheal tube (3.5–4.5 mm), which was clamped to prevent spontaneous breathing. The left jugular vein, brachial artery and femoral artery were catheterised. Ultrasonic flow probes (Transonic Systems) were placed around the left main pulmonary artery, left carotid artery and right femoral artery for continuous recording of blood flows. A near-infrared spectroscopy optode was placed on the head for measurement of regional cerebral oxygenation (crSO_2_, Foresight). Prior to experimentation, lung liquid was drained from the endotracheal tube, which was then re-clamped. Arterial catheters and flow probes were connected to a data acquisition system (Powerlab 16.35, ADInstruments) for continuous real-time physiological measurements, which were digitally recorded on LabChart Pro software at 1000 Hz (ADInstruments v1.8.3) for later offline analysis. Baseline recordings were acquired for 2 minutes prior to lung liquid drainage and experimentation.

Ewes and their foetuses were randomised to two groups: immediate mechanical ventilation for 8 hours (VENT) or asphyxia, resuscitation and mechanical ventilation for 8 hours (ASPHYXIA). While a larger cohort of lambs was included in the physiological analysis, only lambs that had a successful Langendorff procedure were included for all other outcomes. Therefore, the lambs included in this study were:

1. Control_VENT_ (*n*=6; included in all analyses)
2. FGR_VENT_ (*n*=6; included in all analyses)
3. Control_ASPHYXIA_ (*n*=12 for *in vivo* haemodynamic recordings; of which *n*=7 were subsequently used for *ex vivo* Langendorff analyses and associated molecular outcomes)
4. FGR_ASPHYXIA_ (*n*=11 for *in vivo* haemodynamic recordings; of which *n*=6 were subsequently used for *ex vivo* Langendorff analyses and associated molecular outcomes).

Asphyxia was induced by complete umbilical cord occlusion (UCO) whilst withholding respiratory support. The umbilical cord was cut, and the lamb was weighed and transferred to an infant radiant warmer. Asphyxia was continued until the femoral arterial end-diastolic BP declined to ~10 mmHg, which is equivalent to severe asphyxia and bradycardia (<60 bpm). Lambs were initially resuscitated by initiating ventilation in volume-guarantee mode (Babylog 8000+, Dräger) at 7 ml/kg, maximum peak inspiratory pressure (PIP) 35 cmH_2_O, positive end-expiratory pressure (PEEP) 5 cmH_2_O, respiratory rate 60 breaths per minute, inspiratory time 0.3 seconds, and expiratory time 0.7 seconds. This ventilation strategy aligns with the normal tidal volume of spontaneously breathing lambs on continuous positive airway pressure [34]. Resuscitation was deemed successful upon return of spontaneous circulation (ROSC), which was defined as a femoral arterial end-diastolic BP ≥25 mmHg and return of spontaneous pulsatile heart rate (HR) >100 bpm. Asynchronous chest compressions (90 compressions/min) were commenced if 60 seconds of ventilation alone was inadequate in achieving ROSC. Intravenous adrenaline (0.02 to 0.03 mg/kg of 1:10,000 adrenaline; AstraZeneca, Macquarie Park, NSW, Australia) was further administered if lambs failed to achieve ROSC after 60 seconds of chest compressions and ventilation. Anaesthesia was maintained after ROSC through an intravenous infusion of alfaxalone (5–15 mg/kg Alfaxan in 5% glucose).

Lambs were ventilated for 8 hours following ROSC (ASPHYXIA lambs) or upon onset of ventilation (VENT lambs). Regular arterial blood samples were analysed during the ventilation period. The fraction of inspired oxygen (FiO_2_) was initially set at 1.0 and was gradually adjusted to maintain target blood gas values [arterial partial pressure of oxygen (PaO_2_) >40 mmHg, arterial partial pressure of carbon dioxide (PaCO_2_) 35–45 mmHg, pH 7.30–7.45, saturation of oxygen (SaO_2_)>85%]. Ventilation parameters were adjusted to maintain target blood gas values. Dynamic lung compliance, adjusted for birth weight, was calculated by V_T_/ΔP, where ΔP=PIP – PEEP. Ventilator efficiency index (VEI) was calculated by (3800/ΔP × *f* × PaCO_2_), where 3800 ml/kg/min mmHg is the CO_2_ production constant, *f* is the respiratory frequency. The alveolar-arterial difference in oxygen (AaDO_2_) was calculated by [FiO_2_ × (P_atm_ – P_H2O_) – (PaCO_2_/0.8) – PaO_2_], where FiO_2_ is the fraction of inspired oxygen, P_atm_ is the atmospheric pressure (760 mmHg), P_H2O_ is the water vapour pressure at room temperature (47 mmHg at 39°C), 0.8 is the respiratory quotient.

Surfactant (100 mg/kg; Curosurf) was administered after 10 minutes of ventilation. Ewes were humanely killed following delivery of the lamb(s) via an overdose of sodium pentobarbitone (100 mg/kg; Lethabarb, Virbac, Australia). At the end of the ventilation period, lambs underwent euthanasia via cervical dislocation and exsanguination prior to the Langendorff procedure, as outlined in more detail below.

### Langendorff preparation

Immediately before post-mortem, the rate of intravenous alfaxane was doubled to induce anaesthetic overdose, with heparin (i.v. 5000 IU heparin sodium, Pfizer, Victoria, Australia) administered to prevent coronary coagulation. Once deep anaesthesia was confirmed by the absence of a response to web space (metacarpal) compression, euthanasia of the lamb was humanely completed by cervical dislocation and exsanguination before the chest cavity was opened to access the heart. The aorta was clamped, and the heart was swiftly excised and placed in ice-cold, oxygenated Krebs solution. Briefly, Krebs solution contained (in mM) 120 NaCl, 4.7 KCl, 1.2 MgSO_4_·7H_2_O, 1.2 KH_2_PO_4_, 25 NaHCO_3_, 10 glucose, and 1.3 CaCl_2_·H_2_O. The heart was mounted on a Langendorff apparatus via the aorta and constantly perfused with Krebs solution at a constant flow rate of 40 ml/min. A saline-filled latex balloon was inserted into the left ventricle and set to a diastolic pressure of 5 mmHg to measure left ventricular developed pressure (LVDP). Isolated hearts were equilibrated for 10 minutes before baseline recordings were acquired. Measurements of baseline function included coronary perfusion pressure (CPP; measured via pressure ejected from the perfusion line immediately proximal to the aortic cannula), HR, maximal, minimal and mean LVDP, maximal rate of rise of left ventricular contraction (dP/dt_max_), and maximal rate of fall of left ventricular relaxation (dP/dt_min_). Cardiac responses to (i) α_1_-AR stimulation via administration of phenylephrine (10^-5^ to 10^-2^ mmol/l); (ii) β_1_-AR stimulation via administration of dobutamine (10^-7^ to 10^-4^ mmol/l); and (iii) nitric oxide (NO)-mediated vasodilation via administration of glyceryl trinitrate (GTN; 10^-2^ mmol/l) were determined. The heart was allowed to return to baseline function following the completion of each drug curve.

### Tissue collection

Hearts were dismounted at the end of the Langendorff protocol and imaged whole on a stereological grid. Hearts were then transversely sliced into 1-cm-thick sections, with the most superior section of the ventricles incubated in 1% 2,3,5-triphenyltetrahydrozolium chloride (10 mg/ml dissolved in PBS) for 15 minutes at 37°C to determine infarct size. Segments of the left ventricle were either snap-frozen for reverse transcription-quantitative polymerase chain reaction (RT-qPCR) or fixed with 10% neutral-buffered formalin for histological analysis. Cardiac dimensions (length and width) were measured relative to the stereological grid using ImagePro Premier 10 (Media Cybernetics, U.S.A.). Cardiac globularity was quantified by tracing the heart with the polygon tool, and circularity was calculated using the software’s inbuilt measurement tool [Circularity=(4·area)/(π·MaxFeret^2^)], where values closer to one indicate greater globularity.

### RNA extraction and qPCR

Total RNA was extracted from 20 to 30 mg of frozen left ventricular myocardium (RNeasy Mini Kit, Qiagen) before 50 ng/µl of RNA was reverse-transcribed into complementary DNA (cDNA, SuperScript III Reverse Transcriptase, Thermo Fisher). Relative mRNA levels of *α_1_-AR*, *β_1_-AR*, atrial natriuretic peptide (*NPPA*), B-type natriuretic peptide (*NPPB*) and sarcoplasmic/endoplasmic reticulum calcium ATPase 2 (*SERCA/ATP2A2*) (Table 1) were measured by RT-qPCR (QuantStudio 6 Real-Time PCR system, ThermoFisher, U.S.A.). Genes were quantified and normalised to the housekeeping gene 18S using the cycle threshold (ΔC_T_) method. The expression of all genes was expressed relative to the mean of the Control_VENT_ group.

**Table 1:** Primer sequences for RT-qPCR

Gene	Species	Primer sequence	Accession number
18S	Rat	Fwd 5′-GTAACCCGTTGAACCCCATT-3′ Rev 5′-CCATCCAATCGGTAGTAGCG-3′	NR_003286
ɑ_1_-AR	Sheep	Fwd 5′-TGGTCGGCTGCTTCGT-3′ Rev 5′-ACCCAATGGGCATCACTAAGAAA-3′	NC_056055.1
β_1_-AR	Sheep	Fwd 5′-GTGCCCCTGTGCATCATG-3′ Rev 5′-GCAGCTGTCGATCTTCTTCACC-3′	NC_056075.1
NPPA	Sheep	Fwd 5′-CAGTGAAAGGCCAAAGAAGC-3′ Rev 5′-CAGAGCCATACACGGGATTT-3′	NC_056065.1
NPPB	Sheep	Fwd 5′-TGAGCGCCTATCCGCATT-3′ Rev 5′-GAGACATCGGACCCAGAAGTG-3′	NC_056065.1
SERCA/ATP2A2	Sheep	Fwd 5′-GATGTCGCTCCACTTCCTAATC-3′ Rev 5′-TCCATTAGAATCACAGGCAAGG-3′	NC_056070.1

ɑ_1_-AR, ɑ_1_-adrenergic receptor. β_1_-AR, β_1_-adrenergic receptor. NPPA, atrial natriuretic peptide. NPPB, B-type natriuretic peptide. SERCA/ATP2A2, sarcoplasmic/endoplasmic reticulum calcium ATPase 2.

### Histological and immunohistochemical analyses

Left ventricle sections (5 µm thick) were stained with Masson’s trichrome to evaluate perivascular and interstitial fibrosis, as previously described [35]. Ki67 and α_1_-AR immunohistochemistry were performed to identify proliferative cells and α_1_-adrenoceptor populations in left ventricle tissue sections. Tissue sections were washed in PBS (2 × 5 minutes) before antigen retrieval was performed by heating the sections in citric acid buffer (pH 6) in a microwave on high (3 × 10 minutes) followed by 30 minutes cooling in PBS-TX (0.1M, pH 7.4, triton X 1%, 15 minutes). Endogenous peroxidase activity was blocked by immersing the sections in 3% v/v hydrogen peroxide at room temperature (10 minutes), followed by PBS washes (3 × 5 minutes). Sections were incubated in 5% normal goat serum + 2% bovine serum albumin in PBS at room temperature (45 minutes). The primary antibody (Ki67: 1:100 rabbit monoclonal anti-Ki67 antibody, Cat no. MA5-14520, Dako, Glostrup, Denmark; α_1_-adrenoceptor: 1:500 rabbit anti-α_1_-AR antibody, Cat no. OASG00518, Aviva Systems Biology, CA, U.S.A.) was added, and slides were left to incubate at 4°C overnight. Sections were rinsed with PBS (3 × 5 minutes) and incubated with the secondary antibody (biotinylated goat anti-rabbit secondary antibody, 1:200 dilution) in PBS at room temperature (45 minutes) and further rinsed with PBS (3 × 5 minutes). Sections were incubated in strep-horseradish peroxidase (HRP, 1:200 dilution) in PBS at room temperature (45 minutes). Following rinses in PBS (3 × 5 minutes), sections were incubated in 3,3’-Diaminobenzidine (DAB) complex (10 minutes) and rinsed again in PBS (3 × 5 minutes). Slides were lightly counterstained with haematoxylin, dehydrated and coverslipped. Negative control slides confirmed the specificity of Ki67 or α_1_-AR staining. Qualitative assessment of the number of Ki67-positive cells, the area of vascular α_1_-AR staining, and interstitial and perivascular fibrosis was completed by an assessor (ZA) blinded to the experimental groups on coded slides.

### Physiological data analysis

Physiological data were extracted and analysed in LabChart in either 10 seconds epochs every minute (baseline period, asphyxic period and first 15 minutes after ROSC), 30 seconds epochs every 5 minutes (20–60 minutes after ROSC) or 5-minute epochs every hour (1–8 hours after ROSC). As the total duration of asphyxia was not fixed, variables obtained during asphyxia were represented as quartiles relative to the duration of asphyxia for each individual animal. Femoral blood flow was corrected for body weight, while carotid and pulmonary blood flows were corrected for wet brain and lung weights, respectively. Due to missing carotid or femoral blood flow data for some animals, the ratio of carotid to femoral blood flow during asphyxia was derived as a group-level ratio to illustrate overall trends; no statistical analysis was performed for these calculations. The LabChart Blood Pressure module was used to analyse vascular contractility parameters from the femoral BP channel, including the exponential time constant of relaxation (Tau), the maximal rate of rise of contraction (dP/dt_max_), the maximal rate of fall of relaxation (dP/dt_min_), the slope of the isovolumetric relaxation period (IRP) and the contractility index.

### Statistical analysis

Data are expressed as the mean ± standard error of the mean and analysed via GraphPad Prism software (GraphPad Prism 10). Data were assessed for normality using a Shapiro–Wilk test prior to further statistical analysis. Continuous lamb characteristics, baseline heart function, histology and molecular data were analysed via a two-way analysis of variance (ANOVA). Categorical lamb characteristics were analysed using a Chi-square test. Physiology and blood gas measurements were analysed via a three-way repeated measures ANOVA with time, fetal growth (FGR or control) and exposure to asphyxia as fixed variables. *Ex vivo* cardiac responses to drug administration were analysed via a three-way ANOVA with fetal growth (FGR or control), exposure to asphyxia and drug doses as fixed variables. A multiple comparisons uncorrected Fisher’s least significant difference test was conducted to isolate differences in significant interactions between the main factors. Comparisons were deemed statistically significant at a *P*<0.05.

## Results

### Lamb characteristics, asphyxia characteristics and blood gases

Post-mortem lamb characteristics, asphyxia characteristics, baseline blood gases and end-UCO blood gases are described in Table 2. Body weight was significantly lower in FGR lambs compared with control lambs (*P*=0.001). Asymmetric growth restriction was evident with a significantly increased brain-to-body weight ratio (*P*=0.004) and a similar heart-to-body weight ratio. Sex, birth order and ratio of twin to singleton pregnancies were not different between groups. Post-mortem cardiac globularity was reduced in FGR lambs compared with control lambs (*P*=0.014), while heart length and width were similar between groups (Supplementary Table S1).

**Table 2:** Lamb characteristics, asphyxia characteristics and blood gases

Groups	Control_VENT_	FGR_VENT_	Control_ASPHYXIA_	FGR_ASPHYXIA_	*P*-value
*n* (number of lambs)	6	6	12	11	**N/A**
Body weight (kg)	3.1 ± 0.3	2.8 ± 0.1	3.2 ± 0.1	2.4 ± 0.1	***P*_FGR_=0.001** *P*_ASPHYXIA_=0.28 *P*_INT_=0.098
Brain/body weight ratio (g/kg)	14.9 ± 1.4	16.7 ± 0.9	14.0 ± 0.4	17.6 ± 0.9	***P*_FGR_=0.004** *P*_ASPHYXIA_=0.99 *P*_INT_=0.34
Heart/body weight ratio (g/kg)	13.1 ± 0.9	11.1 ± 0.3	11.1 ± 0.4	11.5 ± 0.8	*P*_FGR_=0.33 *P*_ASPHYXIA_=0.29 *P*_INT_=0.13
Sex (% female)	33^†^	33^†^	33	55	*P*=0.10
Birth order (% 1^st^ **)**	67	50	50	55	*P*=0.93
Twin vs. singleton pregnancies (% twins)	100	67	92	100	*P*=0.16
**Baseline blood gases**					
pH	7.29 ± 0.02	7.31 ± 0.02	7.34 ± 0.02	7.32 ± 0.02	*P*_FGR_=0.44 *P*_ASPHYXIA_=0.13 *P*_INT_=0.98
PaO_2_ (mmHg)	23.7 ± 1.4	26.2 ± 4.2	30.9 ± 6.6	22.3 ± 1.7	*P*_FGR_=0.54 *P*_ASPHYXIA_=0.73 *P*_INT_=0.27
PaCO_2_ (mmHg)	49.1 ± 3.2	57.3 ± 2.8	52.2 ± 1.6	54.7 ± 2.1	***P*_FGR_=0.03** *P*_ASPHYXIA_=0.91 *P*_INT_=0.25
SaO_2_ (%)	68.2 ± 3.5	62.3 ± 7.7	69.4 ± 3.9	61.7 ± 5.2	*P*_FGR_=0.20 *P*_ASPHYXIA_=0.94 *P*_INT_=0.86
Haematocrit (%)	38.3 ± 1.6	46.8 ± 3.6*	37.7 ± 0.6	38.2 ± 1.6^#^	***P*_FGR_=0.01** ***P*_ASPHYXIA_=0.01** ***P*_INT_=0.02**
Glucose (mmol/l**)**	1.64 ± 0.16	2.13 ± 0.13*	1.99 ± 0.13	1.59 ± 0.15*^#^	*P*_FGR_=0.76 *P*_ASPHYXIA_=0.54 ***P*_INT_=0.006**
Lactate (mmol/l)	4.29 ± 0.23	5.27 ± 0.80	4.25 ± 0.40	4.71 ± 0.41	*P*_FGR_=0.14 *P*_ASPHYXIA_=0.54 *P*_INT_=0.59
Bicarbonate (mmol/l)	21.8 ± 1.6	24.4 ± 1.5	25.0 ± 1.0	24.8 ± 0.9	*P*_FGR_=0.34 *P*_ASPHYXIA_=0.15 *P*_INT_=0.28
**Asphyxia characteristics**					
Time taken to DBP of 10 mmHg (min)			14.5 ± 0.8	19.2 ± 1.3	***P*=0.005**
Time taken from end-UCO to ROSC (min)			2.4 ± 0.1	2.7 ± 0.3	*P*=0.37
Needed chest compressions (%)			83	82	*P*=0.62
Needed adrenaline bolus (%)			75	55	*P*=0.30
**End-UCO blood gases**					
pH			6.9 ± 0.01	6.9 ± 0.01	*P*=0.89
PaO_2_ (mmHg)			2.4 ± 0.6	6.5 ± 1.9	***P*=0.02**
PaCO_2_ (mmHg)			99.5 ± 6.0	109.2 ± 6.9	*P*=0.30
SaO_2_ (%)			2.6 ± 0.5	3.6 ± 1.2	*P*=0.41
Haematocrit (%)			40.0 ± 2.0	40.0 ± 1.8	*P*=0.97
Lactate (mmol/l)			9.12 ± 0.9	10.1 ± 0.5	*P*=0.35
Bicarbonate (mmol/l)			13.4 ± 0.8	14.1 ± 0.5	*P*=0.50

Data expressed as mean ± standard error of the mean and analysed by 2-way ANOVA with a Fisher’s least significant difference multiple comparisons test (lamb characteristics and baseline blood gases), an unpaired Student’s t-test (asphyxia characteristics and end-UCO blood gases) or a Chi-squared test (sex and birth order). Statistical significance threshold *P<0.05 FGR vs. control; ^#^ *P*<0.05 asphyxia vs. ventilation. ^†^Signifies exclusion of the sex of two lambs due to ambiguous genitalia.

DBP, diastolic blood pressure;. FGR, fetal growth restriction. ROSC, return of spontaneous circulation. UCO, umbilical cord occlusion.

FGR groups were hypercapnic at baseline compared with control groups (*P*=0.03). FGR_VENT_ lambs had significantly higher haematocrit levels than Control_VENT_ lambs (*P*=0.02), while FGR_ASPHYXIA_ lambs had significantly lower haematocrit levels than FGR_VENT_ lambs (*P*=0.009). Baseline glucose levels of FGR_VENT_ lambs were significantly higher than Control_VENT_ lambs (*P*=0.047), while FGR_ASPHYXIA_ lambs had significantly lower glucose levels compared with FGR_VENT_ (*P*=0.02) and Control_ASPHYXIA_ (*P*=0.04) lambs. No other differences in baseline arterial blood gas parameters were evident.

FGR_ASPHYXIA_ lambs took significantly longer to reach an end-diastolic BP of 10 mmHg compared with Control_ASPHYXIA_ lambs (14.5 ± 0.8 min vs. 19.2 ± 1.3 min; *P*=0.005). However, the time taken to achieve ROSC did not differ between groups. The requirements for resuscitation interventions (chest compressions and adrenaline boluses) were not different between groups.

At the end of the asphyxia period, arterial pH, PaCO_2_, SaO_2_, haematocrit, lactate and bicarbonate were not different between Control_ASPHYXIA_ and FGR_ASPHYXIA_ lambs. However, Control_ASPHYXIA_ lambs had significantly lower PaO_2_ at the end of the asphyxia period compared with FGR_ASPHYXIA_ lambs (*P*=0.02).

### Physiology during asphyxia

We have previously characterised the real-time physiological response to asphyxia [27], which is also represented in Supplementary Figure S1. However, as the duration of asphyxia significantly differed between groups (Table 2) and real-time data are influenced by varying attrition rates, the physiological responses to asphyxia are presented here as quartiles relative to each lamb’s individual duration of asphyxia.

**Figure 1:**
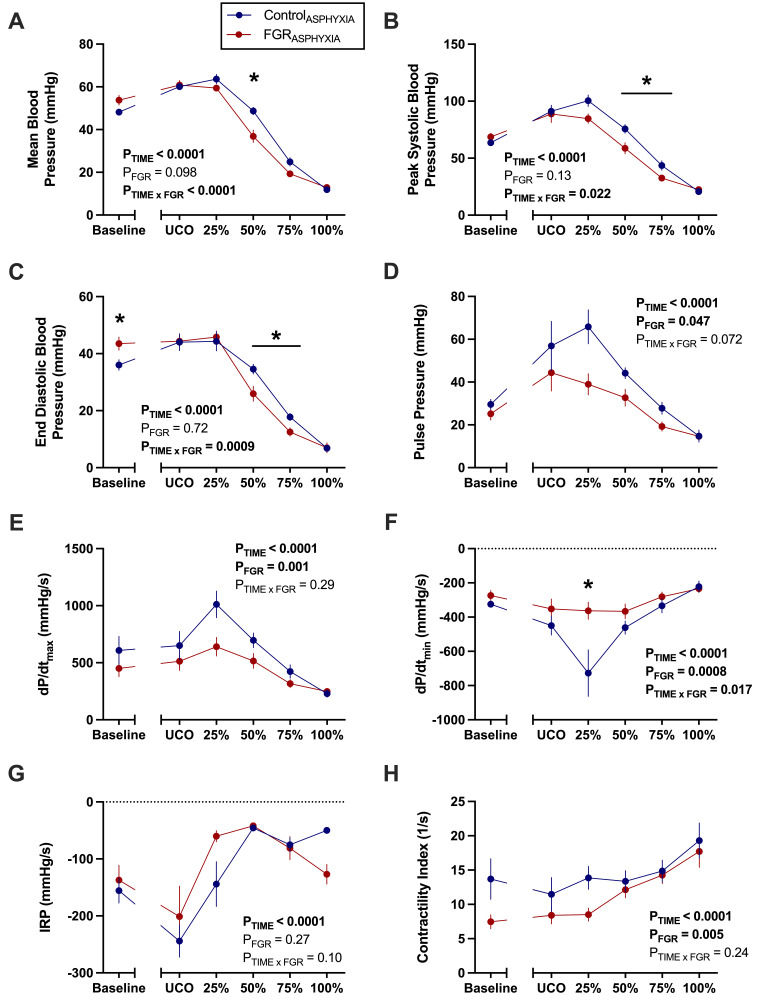
Blood pressure and vascular contractility during asphyxia. Data presented as mean ± standard error of the mean of (**A**) mean arterial blood pressure, (**B**) peak systolic blood pressure, (**C**) end diastolic blood pressure, (**D**) pulse pressure, (**E**) dP/dt_max_, (**F**) dP/dt_min_, (**G**) isovolumetric relaxation period (IRP) and (**H**) contractility index. Data are presented at baseline, umbilical cord occlusion (UCO), and at quartiles relative to the total duration of asphyxia (25, 50, 75 and 100%). Groups are asphyxiated control (Control_ASPHYXIA_, *n*=12) and asphyxiated FGR (FGR_ASPHYXIA_, *n*=11) lambs. Data were analysed via a repeated measure mixed effects analysis with Fisher’s least significant difference multiple comparisons test. *P<0.05.

Mean arterial BP was significantly lower in FGR_ASPHYXIA_ lambs at 50% asphyxia compared with Control_ASPHYXIA_ lambs (*P*=0.02; Figure 1A). Peak systolic and end diastolic BPs were significantly lower in FGR_ASPHYXIA_ lambs compared with Control_ASPHYXIA_ lambs between 50%–75% of the asphyxic period (*P*<0.05; Figure 1B&C). FGR_ASPHYXIA_ lambs had significantly lower pulse pressures compared with Control_ASPHYXIA_ lambs during asphyxia (*P*=0.047; Figure 1D). Mean HR was not different between groups during asphyxia (data not shown).

Vascular contractility parameters were also derived from the arterial pressure signal (Figure 1E–H). FGR_ASPHYXIA_ lambs had a significantly lower dP/dt_max_, dP/dt_min_ and contractility index compared with Control_ASPHYXIA_ lambs throughout the asphyxic period (*P*<0.05; Figure 1E, F and H). Control_ASPHYXIA_ lambs had a steeper decline in dP/dt_min_ (*P*<0.0001; Figure 1F) compared with FGR_ASPHYXIA_ lambs at 25% asphyxia.

**Figure 2:**
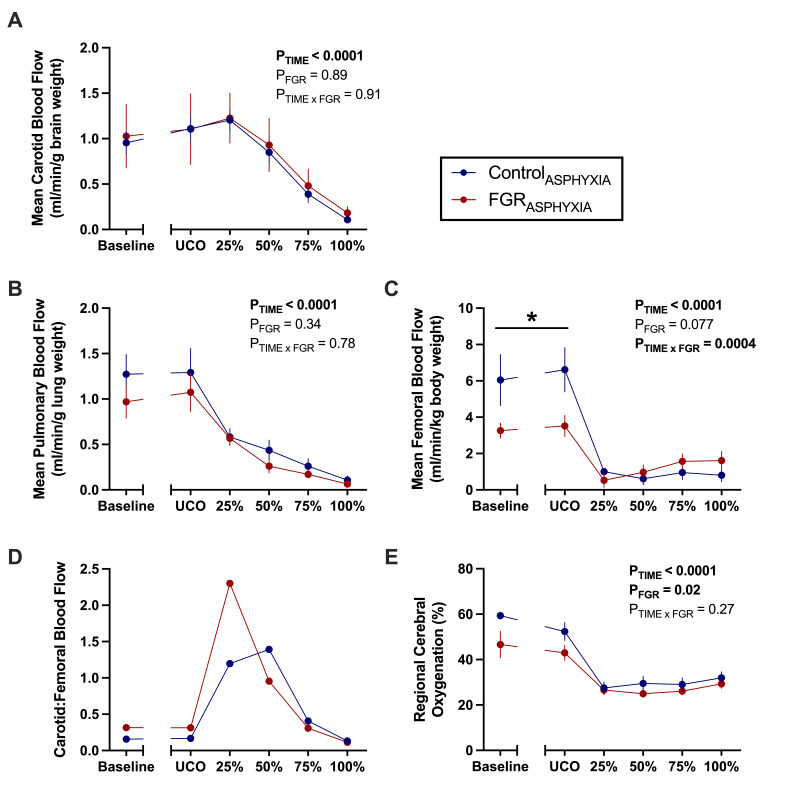
Blood flows and regional cerebral oxygenation during asphyxia. Data presented as mean ± standard error of the mean of (**A**) mean carotid blood flow (corrected for brain weight), (**B**) mean pulmonary blood flow (corrected for lung weight), (**C**) mean femoral blood flow (corrected for body weight), (**D**) ratio of carotid to femoral blood flow and (**E**) mean regional cerebral oxygenation. Data are presented at baseline, umbilical cord occlusion (UCO), and at quartiles relative to the total duration of asphyxia (25, 50, 75 and 100%). Groups are asphyxiated control (Control_ASPHYXIA_, *n*=12) and asphyxiated FGR (FGR_ASPHYXIA_, *n*=11) lambs. Data were analysed via repeated measure mixed effects analysis with Fisher’s least significant difference multiple comparisons test. *P<0.05. Due to occasional missing carotid or femoral measurements, the ratio of carotid to femoral blood flow (**D**) was calculated from group mean flows for visualisation only; no error bars or statistical analyses were performed for this parameter.

Carotid and pulmonary blood flows were not different between Control_ASPHYXIA_ and FGR_ASPHYXIA_ lambs during asphyxia (Figure 2A&B). However, femoral blood flow was significantly lower in FGR_ASPHYXIA_ than Control_ASPHYXIA_ at baseline and immediately after UCO (*P*<0.05; Figure 2C). Carotid to femoral blood flow ratio was not different between groups during asphyxia (Figure 2D). FGR_ASPHYXIA_ lambs had a significantly lower regional cerebral oxygenation during asphyxia compared with Control_ASPHYXIA_ lambs (*P*=0.02; Figure 2E).

### Blood gases during ventilation

**Figure 3:**
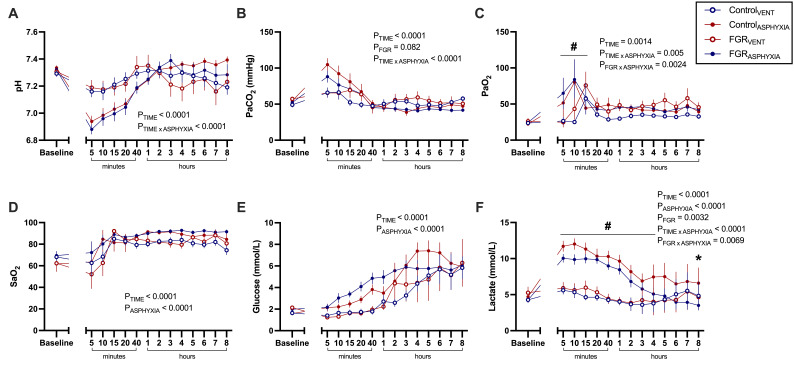
Arterial blood gas parameters during ventilation. Data presented as mean ± standard error of the mean of (**A**) pH, (**B**)arterial partial pressure of carbon dioxide (PaCO_2_), (**C**) arterial partial pressure of oxygen (PaO_2_), (**D**) saturation of oxygen (SaO_2_), (**E**) glucose and (**F**) lactate at baseline and during 8 hours of post-recovery ventilation. Groups are ventilated control (Control_VENT_, *n*=6), ventilated FGR (FGR_VENT_, *n*=6), asphyxiated control (Control_ASPHYXIA_, *n*=12) and asphyxiated FGR (FGR_ASPHYXIA_, *n*=11) lambs. Data were analysed by a repeated measure mixed effects analysis with a Fisher’s least significant difference multiple comparisons test. *P<0.05 FGR vs. control; ^#^ *P*<0.05 asphyxia vs. ventilation.

Arterial pH, PaCO_2_, SaO_2_ and glucose levels were similar between groups during ventilation (Figure 3A&B and D&E). Control_ASPHYXIA_ lambs had significantly higher PaO_2_ compared with Control_VENT_ animals at 10 and 15 minutes after ROSC (*P*<0.05; Figure 3C). FGR_ASPHYXIA_ lambs had a significantly lower PaO_2_ than FGR_VENT_ lambs at 10 minutes, but this rebounded, resulting in a significantly higher PaO_2_ than FGR_VENT_ lambs at 15 minutes (*P*<0.05; Figure 3C). Lactate concentrations were significantly higher in Control_ASPHYXIA_ lambs compared with Control_VENT_ lambs from 5 minutes to 2 hours after ROSC (*P*<0.05; Figure 3F). Lactate concentrations were also significantly higher in FGR_ASPHYXIA_ lambs compared with FGR_VENT_ animals between 5 minutes and 2 hours and at 4 hours after ROSC (*P*<0.05; Figure 3F). FGR_ASPHYXIA_ lambs also had significantly higher lactate levels at 8 hours after ROSC compared with Control_ASPHYXIA_ lambs (*P*=0.03; Figure 3F).

### Ventilatory parameters

Dynamic lung compliance and AaDO_2_ were similar between groups during ventilation (Supplementary Figure S2A&C). The VEI was higher in asphyxiated lambs compared with ventilated lambs in the final half of ventilation (Supplementary Figure S2B).

### Physiology during ventilation

One FGR_ASPHYXIA_ lamb developed a pneumothorax approximately 2.5 hours after the onset of ventilation and was excluded from analysis. No lambs from other groups developed pneumothoraces during ventilation.

#### Blood pressure and vascular contractility

**Figure 4:**
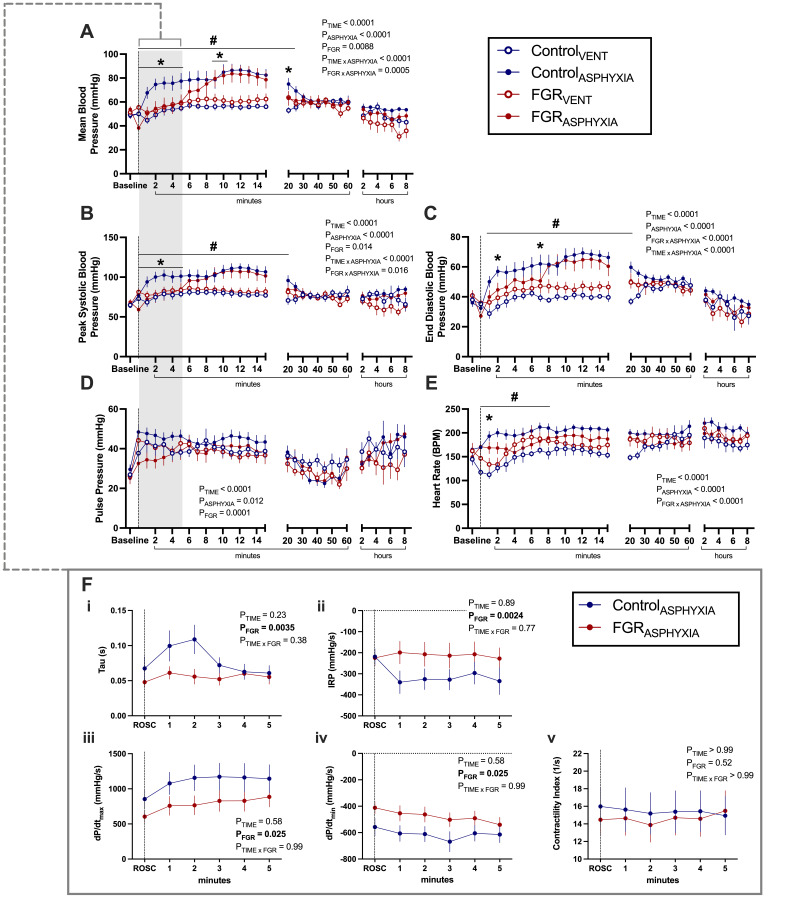
Blood pressure and vascular contractility during ventilation. Data presented as mean ± standard error of the mean of (**A**) mean blood pressure, (**B**) peak systolic blood pressure, (**C**) end diastolic blood pressure, (**D**) pulse pressure, (**E**) mean heart rate, and (**F**) blood pressure-derived vascular contractility in the first 5 minutes after ROSC, including (**i**) Tau, (**ii**) isovolumetric relaxation period (IRP), (**iii**) dP/dt_max_, (**iv**) dP/dt_min_ and (**v**) contractility index. Data are presented at baseline, at ROSC (┄ dotted line), and during 8 hours of post-recovery ventilation. Groups are ventilated control (Control_VENT_, *n*=6), ventilated FGR (FGR_VENT_, *n*=6), asphyxiated control (Control_ASPHYXIA_, *n*=12) and asphyxiated FGR (FGR_ASPHYXIA_, *n*=11) lambs. Data were analysed by a repeated measure mixed effects analysis with a Fisher’s least significant difference multiple comparisons test. *P<0.05 FGR vs. control; ^#^ *P*<0.05 asphyxia vs. ventilation.

BP, HR and pulse pressure after ROSC and during mechanical ventilation are shown in Figure 4. At ROSC, FGR_ASPHYXIA_ lambs had a significantly lower mean arterial BP than Control_ASPHYXIA_ (*P*=0.048) and FGR_VENT_ lambs (*P*=0.02; Figure 4A). Control_ASPHYXIA_ lambs had a significantly higher mean arterial BP compared with Control_VENT_ lambs for the first 20 minutes after ROSC (*P*<0.05; Figure 4A) and compared with FGR_ASPHYXIA_ lambs for the first 5 minutes after ROSC (*P*<0.05; Figure 4A). However, the mean arterial BP of FGR_ASPHYXIA_ lambs began to rise after 5 minutes and was significantly higher than FGR_VENT_ lambs between 9 and 15 minutes after ROSC (*P*<0.05; Figure 4A). This rebound relative hypertension was transient in FGR_ASPHYXIA_ lambs and returned to a significantly lower mean arterial BP compared with Control_ASPHYXIA_ lambs by 20 minutes (Figure 4A; *P*=0.049).

At ROSC, the systolic BP of FGR_ASPHYXIA_ lambs was significantly lower than FGR_VENT_ (*P*=0.01) and Control_ASPHYXIA_ lambs (*P*=0.02; Figure 4B). Systolic BP remained significantly higher in Control_ASPHYXIA_ lambs compared with FGR_ASPHYXIA_ lambs for the first 10 minutes after ROSC (*P*<0.05; Figure 4B) and compared with Control_VENT_ lambs for the first 20 minutes after ROSC (*P*<0.05; Figure 4B). The increase in systolic BP was delayed in FGR_ASPHYXIA_ lambs and was only demonstrated after 10 minutes, with significantly higher pressures compared with FGR_VENT_ lambs between 10 to 15 minutes after ROSC (*P*<0.05; Figure 4B).

End diastolic BPs were similar between groups at ROSC. Between 1 and 25 minutes, Control_ASPHYXIA_ lambs had significantly higher end diastolic BPs compared with Control_VENT_ lambs (*P*<0.05; Figure 4C) and at 2 minutes and 7 minutes after ROSC compared with FGR_ASPHYXIA_ lambs (*P*<0.05; Figure 4C). FGR_ASPHYXIA_ lambs also had significantly lower end diastolic BP compared with FGR_VENT_ lambs between 8 and 15 minutes (*P*<0.05; Figure 4C).

Mean, systolic and diastolic BP levels remained similar between groups from 30 minutes until the end of the ventilation period.

The HR of Control_ASPHYXIA_ lambs was significantly higher than Control_VENT_ lambs at ROSC and for the first 8 minutes after ROSC (*P*<0.05; Figure 4E) and compared with FGR_ASPHYXIA_ lambs between 2 and 7 minutes after ROSC (*P*<0.05; Figure 4E). FGR_ASPHYXIA_ lambs had a significantly higher HR compared with FGR_VENT_ lambs 1 minute after ROSC (*P*=0.046; Figure 4E). Pulse pressures were not different between groups at ROSC or during the ventilation period (Figure 4D).

The vascular contractility of Control_ASPHYXIA_ and FGR_ASPHYXIA_ lambs in the first 5 minutes after ROSC was further investigated via the arterial BP waveform (Figure 4F). In the first 5 minutes after ROSC, Tau, IRP, dP/dt_max_ and dP/dt_min_ were significantly lower in FGR_ASPHYXIA_ lambs compared with Control_ASPHYXIA_ lambs (*P*<0.05; Figure 4F[i–iv]). However, the contractility index was not different between groups during this period (Figure 4F[v]).

#### Blood flows and regional cerebral oxygenation

**Figure 5:**
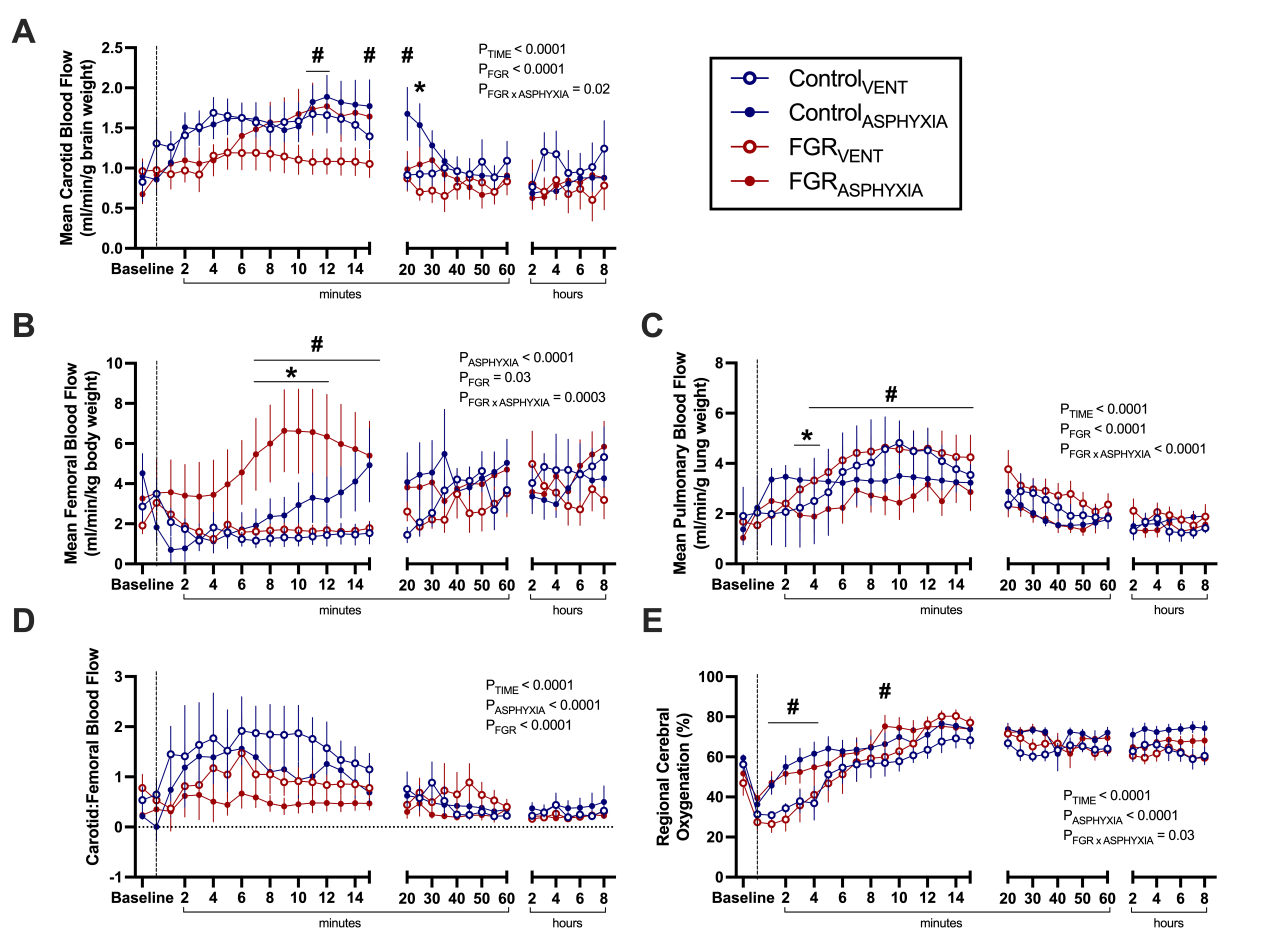
Blood flows and regional cerebral oxygenation during ventilation. Data presented as mean ± standard error of the mean of (**A**) mean carotid blood flow (corrected for brain weight), (**B**) mean femoral blood flow (corrected for body weight), (**C**) mean pulmonary blood flow (corrected for lung weight), (**D**) ratio of carotid to femoral blood flow and (**E**) mean regional cerebral oxygenation. Data are presented at baseline, at return of spontaneous circulation (┄ dotted line), and during 8 hours of post-recovery ventilation. Groups are ventilated control (Control_VENT_, *n*=6), ventilated FGR (FGR_VENT_, *n*=6), asphyxiated control (Control_ASPHYXIA_, *n*=12) and asphyxiated FGR (FGR_ASPHYXIA_, *n*=11) lambs. Data analysed via a repeated measure mixed effects analysis with a Fisher’s least significant difference multiple comparisons test. *P<0.05 FGR vs. control; ^#^ *P*<0.05 asphyxia vs. ventilation.

Mean carotid blood flow was higher in FGR_ASPHYXIA_ compared with FGR_VENT_ lambs between 11 and 12 minutes after ROSC (*P*<0.05; Figure 5A). Mean carotid blood flow was also higher in Control_ASPHYXIA_ compared with Control_VENT_ lambs at 20 and 25 minutes (*P*<0.05; Figure 5A) and compared with FGR_ASPHYXIA_ lambs at 25 minutes (*P*=0.008; Figure 5A).

Mean femoral blood flow was higher in FGR_ASPHYXIA_ compared with Control_ASPHYXIA_ lambs between 7 and 12 minutes after ROSC (*P*<0.05; Figure 5B) and compared with FGR_VENT_ lambs between 7 and 15 minutes after ROSC (*P*<0.05; Figure 5B). The ratio of carotid to femoral blood flow was not different between groups after ROSC (Figure 5D).

Mean pulmonary blood flow was lower in FGR_ASPHYXIA_ lambs compared with Control_ASPHYXIA_ lambs between 3 and 4 minutes after ROSC (*P*<0.05; Figure 5C) and compared with FGR_VENT_ lambs between 4 and 15 minutes after ROSC (*P*<0.05; Figure 5C).

Regional cerebral oxygenation was higher in FGR_ASPHYXIA_ lambs compared with FGR_VENT_ lambs from 1 to 3 minutes and at 9 minutes after ROSC (*P*<0.05; Figure 5E). Regional cerebral oxygenation was also higher in Control_ASPHYXIA_ lambs compared with Control_VENT_ lambs between 2 and 4 minutes after ROSC (*P*<0.05; Figure 5E).

### Langendorff perfusion

#### Baseline *ex vivo* function

Isolated lamb hearts were assessed via a Langendorff perfusion to understand the isolated role of cardiac function in response to a secondary asphyxia. Basal isolated cardiac function, including CPP, HR, mean and maximal LVDP amplitude, and the maximal rate of rise of contraction (dP/dt_max_) and relaxation (dP/dt_min_), was similar between groups (Supplementary Figure S3).

#### Response to α_1_-adrenergic stimulation via phenylephrine

**Figure 6:**
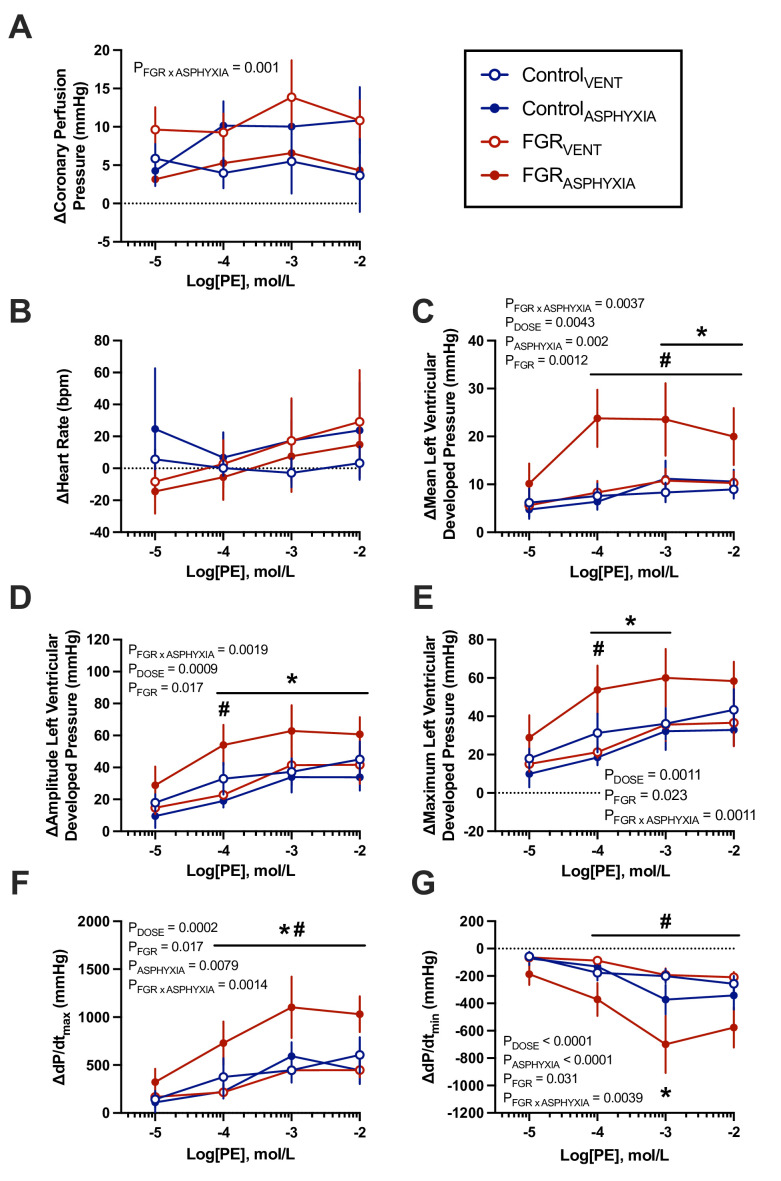
*Ex vivo* cardiac responses to phenylephrine. Data presented as mean ± standard error of the mean change in (**A**) coronary perfusion pressure, (**B**) heart rate, (**C**) mean left ventricular developed pressure, (**D**) amplitude of left ventricular developed pressure, (**E**) maximum left ventricular developed pressure, (**F**) maximal left ventricular contractility (dP/dt_max_) and (**G**) minimal left ventricular contractility (dP/dt_min_) in response to increasing concentrations of phenylephrine. Groups are ventilated control (Control_VENT_, *n*=6), ventilated FGR (FGR_VENT_, *n*=6), asphyxiated control (Control_ASPHYXIA_, *n*=7), and asphyxiated FGR (FGR_ASPHYXIA_, *n*=6) lambs. Data were analysed via mixed effects analysis with uncorrected Fisher’s least significance multiple comparisons test; statistical significance threshold *P*<0.05. *P<0.05 FGR vs. control; ^#^ *P*<0.05 asphyxia vs. ventilation.

Hearts from FGR_ASPHYXIA_ animals showed greater increases in mean LVDP, dP/dt_max_ and dP/dt_min_ compared with FGR_VENT_ animals in response to 10^-4^ to 10^-2^ doses of phenylephrine (*P*<0.05; Figure 6C and F&G). FGR_ASPHYXIA_ animals also had greater increases in mean LVDP compared with Control_ASPHYXIA_ animals in response to 10^-3^ and 10^-2^ doses of phenylephrine (*P*<0.05; Figure 6C). FGR_ASPHYXIA_ hearts also demonstrated greater increases in LVDP amplitude (10^-4^ to 10^-2^ doses) and maximal LVDP (10^-4^ to 10^-3^ doses) compared with Control_ASPHYXIA_ hearts in response to phenylephrine administration (*P*<0.05; Figure 6D&E). FGR_ASPHYXIA_ hearts also had a greater amplitude and maximal LVDP response compared with FGR_VENT_ hearts following administration of the 10^-4^ dose of phenylephrine (*P*<0.05; Figure 6D&E). FGR_ASPHYXIA_ hearts experienced greater increases in dP/dt_max_ (10^-4^ to 10^-2^ dose) and dP/dt_min_ (10^-3^ dose) in response to phenylephrine compared with Control_ASPHYXIA_ animals (*P*<0.05; Figure 6F&G). HR responses to phenylephrine were not different between groups (Figure 6B).

#### Response to β_1_-adrenergic stimulation via dobutamine

**Figure 7:**
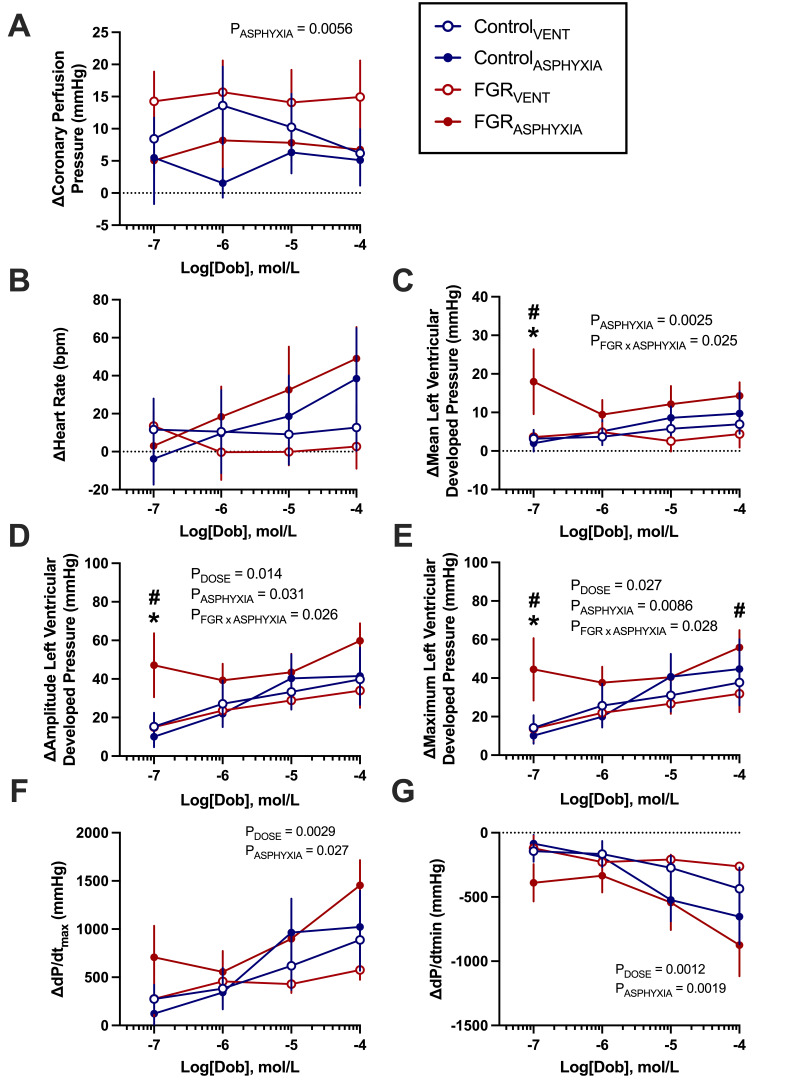
*Ex vivo* cardiac responses to dobutamine. Data presented as mean ± standard error of the mean change in (**A**) coronary perfusion pressure, (**B**) heart rate, (**C**) mean left ventricular developed pressure, (**D**) amplitude of left ventricular developed pressure, (**E**) maximum left ventricular developed pressure, (**F**) maximal left ventricular contractility (dP/dt_max_) and (**G**) minimal left ventricular contractility (dP/dt_min_) in response to increasing concentrations of dobutamine. Groups are ventilated control (Control_VENT_, *n*=6), ventilated FGR (FGR_VENT_, *n*=6), asphyxiated control (Control_ASPHYXIA_, *n*=7), and asphyxiated FGR (FGR_ASPHYXIA_, *n*=6) lambs. Data were analysed via mixed effects analysis with uncorrected Fisher’s least significance multiple comparisons test; statistical significance threshold *P*<0.05. *P< 0.05 FGR vs. control; ^#^ *P*<0.05 asphyxia vs. ventilation.

Hearts from ventilated lambs experienced a greater increase in CPP in response to dobutamine compared with hearts from asphyxiated lambs (*P*=0.006; Figure 7A). FGR_ASPHYXIA_ hearts had a greater increase in mean, amplitude and maximal LVDP following a 10^-7^ dose of dobutamine compared with Control_ASPHYXIA_ and FGR_VENT_ hearts (*P*<0.05; Figure 7B–E). FGR_ASPHYXIA_ hearts also had a greater increase in maximal LVDP compared with FGR_VENT_ hearts following a 10^-4^ dose of dobutamine (*P*=0.049; Figure 7E). Asphyxiated hearts also demonstrated a larger increase in dP/dt_max_ and dP/dt_min_ following dobutamine (*P*<0.05; Figure 7F&G). HR responses following dobutamine were not different between groups (Figure 7B).

#### Response to vasodilator via glyceryl trinitrate

Isolated cardiac responses to GTN, a NO donor, were not different between groups (Supplementary Figure S4).

### Histology

**Figure 8:**
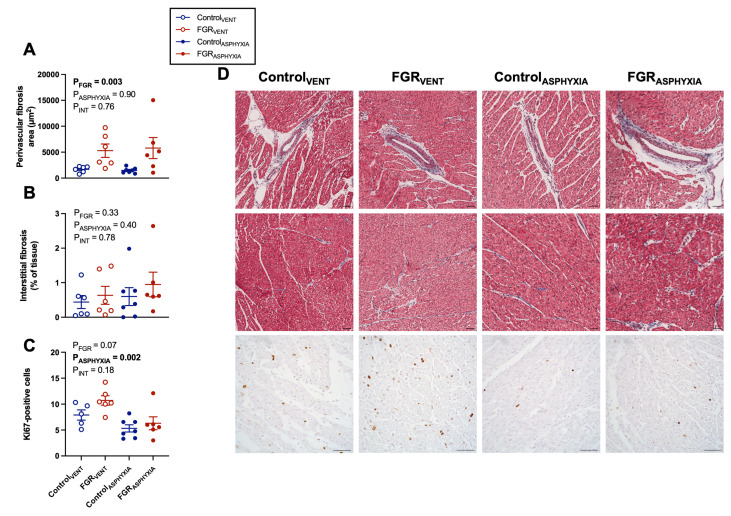
Fibrosis area and cell proliferation in the left ventricle. Data presented as mean ± standard error of (**A**) perivascular fibrosis area, (**B**) % of interstitial fibrosis (relative to tissue area), and (**C**) number of proliferative (Ki67-positive) cells in the left ventricle. (**D**) Representative photomicrographs of Masson’s trichrome-stained left ventricle to evaluate perivascular and interstitial fibrosis and Ki67-positive cells evaluating cell proliferation. Groups are ventilated control (Control_VENT_, *n*=6), ventilated FGR (FGR_VENT_, *n*=6), asphyxiated control (Control_ASPHYXIA_, *n*=7), and asphyxiated FGR (FGR_ASPHYXIA_, *n*=6) lambs. Data were analysed via two-way ANOVA; statistical significance threshold *P*<0.05. Scale bar=50 µm.

Histological assessment of the left ventricle was undertaken to assess the cardiac pathology associated with FGR and asphyxia. Perivascular fibrosis area, but not interstitial fibrosis, was significantly higher in FGR groups compared with controls (*P*=0.003; Figure 8A&B). The number of Ki67-positive cells was significantly lower in asphyxia groups (*P*=0.002; Figure 8C). Ventricular gross infarct size and left ventricular vascular ɑ_1_-AR content were not different between groups (Table 3 and Supplementary Figure S5).

**Table 3:** Histological and RT-qPCR analysis.

	Control_VENT_	FGR_VENT_	Control_ASPHYXIA_	FGR_ASPHYXIA_	*P*-value
**Histology**					
Ventricular gross infarct size (% relative to total tissue area)	7.4 ± 1.3	9.2 ± 3.7	11.6 ± 3.4	9.9 ± 1.3	*P*_FGR_=0.99 *P*_ASPHYXIA_=0.33 *P*_INT_=0.47
Ventricular gross infarct (% increase relative to Control_VENT_)	0 ± 17.9	24.2 ± 49.3	56.4 ± 45.7	32.9 ± 17.9	*P*_FGR_=0.99 *P*_ASPHYXIA_=0.33 *P*_INT_=0.47
**RT-qPCR (Left ventricle)**					
ɑ_1_-AR	1.0 ± 0.1	1.3 ± 0.3	0.9 ± 0.2	1.2 ± 0.4	*P*_FGR_=0.31 *P*_ASPHYXIA_=0.79 *P*_INT_=0.96
β_1_-AR	1.0 ± 0.1	3.3 ± 1.6	3.0 ± 0.7	1.9 ± 0.4	*P*_FGR_=0.51 *P*_ASPHYXIA_=0.79 *P*_INT_=0.08
NPPA	1.0 ± 0.4	1.4 ± 0.5	1.5 ± 0.6	0.9 ± 0.2	*P*_FGR_=0.77 *P*_ASPHYXIA_=0.99 *P*_INT_=0.28
NPPB	1.0 ± 0.5	0.2 ± 0.08	0.4 ± 0.1	1.7 ± 0.8*^#^	*P*_FGR_=0.53 *P*_ASPHYXIA_=0.38 ***P*_INT_=0.03**
SERCA/ATP2A2	1.0 ± 0.2	2.1 ± 0.5	1.8 ± 0.2	2.3 ± 0.3	***P*_FGR_=0.02** *P*_ASPHYXIA_=0.11 *P*_INT_=0.40

Data shown as mean ± SEM and analysed via a two-way ANOVA with a Fisher’s least significant difference multiple comparisons test. RT-qPCR results expressed as a fold change from the mean of the Control_VENT_ group. *P< 0.05 FGR vs. control; ^#^ *P*<0.05 asphyxia vs. ventilation.

ɑ_1_-AR, ɑ_1_-adrenergic receptor. β_1_-AR, β_1_-adrenergic receptor. NPPA, atrial natriuretic peptide. NPPB, B-type natriuretic peptide. SERCA/ATP2A2, sarcoplasmic/endoplasmic reticulum calcium ATPase 2.

### Gene expression

The mRNA expression of *ɑ_1_-AR*, *β_1_-AR* and *NPPA* in the left ventricle was not different between groups (Table 3). The mRNA expression of *NPPB* was higher in FGR_ASPHYXIA_ animals compared with Control_ASPHYXIA_ (*P*=0.049) and FGR_VENT_ (*P*=0.03) groups (Table 3). The mRNA expression of *SERCA/ATP2A2* was also higher in FGR groups compared with controls (*P*=0.02; Table 3).

## Discussion

FGR and perinatal asphyxia both independently increase the risk of cardiovascular disease [4,36], yet their combined effects on cardiovascular function during the perinatal period remain poorly understood. Additionally, preterm birth may further exacerbate lifelong cardiovascular risks in FGR neonates delivered early to mitigate the risk of stillbirth [31,32,37]. In this study, we examined the cardiovascular responses of preterm control and FGR lambs to a severe asphyxic insult around the time of birth. Isolated hearts from FGR lambs demonstrated alterations in α_1_- and β_1_-AR function independent of adrenergic receptor abundance, suggesting potential compensatory mechanisms involving sympathetic activation to maintain cardiac contractility during a period of asphyxia. We showed that despite FGR lambs having an ability to withstand asphyxia for longer, they could not adequately maintain BP after recovery from asphyxia due to poor vascular and cardiac contractility. These distinct vascular and cardiac adaptations highlight how antenatal complications may affect an infant’s response to perinatal asphyxia.

We previously demonstrated that FGR lambs are more resilient to a mild asphyxic event [27], suggesting that exposure to chronic hypoxia *in utero*, such as in those diagnosed with FGR, results in unique adaptations to an additional asphyxic insult. Here, we extended these findings by increasing the severity of asphyxia and found that FGR lambs withstood asphyxia for an average of 5 minutes longer than control lambs. However, FGR lambs had consistently lower BPs and a reduced capacity to maintain cerebral oxygenation during asphyxia, suggesting that the increased duration of asphyxia is a critical dampening of the baroreflex-autonomic system and should not be interpreted as true ‘resilience’ to a secondary insult. The normal fetal response to profound asphyxia is characterised by a sharp increase in sympathetic activity and subsequent increase in BP, initially activated by α-adrenergic afferents [38], to maintain perfusion to critical organs through the baroreflex response [25]. Although FGR and control lambs had similar carotid-to-femoral blood flow ratios, indicating comparable abilities in maintaining blood flow to the brain, only control lambs mounted an early and robust cardiac response to asphyxia. In contrast, FGR lambs demonstrated impairments in the vascular and cardiac response to asphyxia, characterised by lower BPs, lower pulse pressure and blunted maximal rates of contraction and relaxation within the cardiovascular system. Impaired vascular and cardiac contractility increases the risk of cerebral and coronary hypoperfusion [39]. Pulse pressure, a robust indicator of left ventricular output in neonates [40], was significantly lower in FGR lambs compared with controls, a finding likely influenced by the abnormalities in contractility within the cardiovascular system. Impaired cardiac contractility in FGR lambs likely reflects underlying cardiac remodelling, which is often associated with increased cardiac globularity in preterm FGR infants [12,41]. Notably, preterm birth itself can also independently contribute to increased cardiac globularity [42]. We observed reduced globularity in our FGR cohort, in contrast to previous preclinical studies [43], indicating *in utero* cardiac remodelling into a more elongated shape [44,45]. Additionally, we observed excess perivascular collagen deposition in FGR lambs, which can impede force transduction between cardiomyocytes, thereby reducing myocardial contractility and contributing to systolic and diastolic impairments [46]. Reduced cardiac contractility may also result from downstream molecular alterations, as shown in FGR fetal sheep induced by placental carunclectomy that demonstrated decreased phosphorylation of cardiac troponin, a key regulator of myofilament calcium sensitivity [47], and metabolic remodelling of the left ventricle [48]. Previous *in utero* sheep studies report conflicting evidence on whether FGR foetuses exhibit enhanced [28,49,50] or diminished [25,26] BP maintenance following a secondary hypoxic or hypotensive insult. However, these previous studies were conducted with the placenta still supporting circulatory function, whereas in our study, FGR lambs had a reduced capacity to sustain cardiovascular stability once placental support was removed. Taken together, poor vascular and cardiac function contributes to the failure of FGR lambs to dynamically alter and maintain BP during asphyxia, potentially increasing the risk of circulatory instability in the perinatal period.

Asphyxiated neonates are at increased risk of haemodynamic instability following the restoration of cardiac output, which can contribute to multi-organ failure and metabolic dysregulation [2]. In this study, FGR lambs demonstrated a delayed hypertensive overshoot in BP compared with control lambs immediately after ROSC. Rebound hypertension leads to cerebrovascular injury in the neonate [51]. In term lambs, rebound hypertension after ROSC increases blood vessel protein extravasation in the white and grey matter of the brain, which is associated with the loss of tight junction proteins regulating vascular integrity [52]. Preterm FGR infants may be more sensitive to sudden fluctuations in cardiac afterload, leading to an increased risk of cardiovascular and cerebrovascular injury. In particular, low cardiac output in the first 24 hours of life results in increased risk of hypoperfusion-reperfusion brain injury in very low birth weight infants [53]. However, the susceptibility of asphyxiated FGR lambs to increased neuropathology remains unknown, as brain histopathology was not examined in the current study. Fluctuating myocardial perfusion is also likely to cause injury to the FGR heart, as previous studies have shown that isolated hearts from preterm FGR lambs present with larger infarct areas following ischaemia-reperfusion injury [54,55]. This suggests the potential for an increased risk of cardiac injury following a secondary insult in the FGR heart, although this was not evident in the current study, where comparable levels of myocardial infarction were observed in FGR and control groups. It is possible that our model either induced a less severe myocardial injury than complete asystole or ischaemia-reperfusion injury, or an inadequate duration of exposure to a hypoxic injury. However, asphyxiated FGR lambs had a higher left ventricular mRNA expression of NPPB, a marker of increased ventricular pressure and wall stress [56]. Although not assessed in the current study, impairments in cardiac metabolism in FGR, including lower glycogen reserves, reduced glucose utilisation and mitochondrial dysfunction [57,58], may also contribute to poorer cardiovascular outcomes following asphyxia. During asphyxia, the acute decline in oxygen induces a shift from oxidative phosphorylation to anaerobic glycolysis [59], while catecholamine release mobilises hepatic and muscular glycogen stores to generate ATP [60]. Consequently, a depletion of glycogen stores can reduce glucose availability, impair anaerobic glycolysis and promote lactate accumulation and subsequent metabolic acidosis [60]. Overall, this suggests that FGR hearts may be more susceptible to subclinical ventricular injury following asphyxia despite having similar infarct sizes to asphyxiated controls.

In the current study, we found that the isolated hearts of asphyxiated FGR lambs were significantly more contractile in response to α_1_-adrenergic activation compared with asphyxiated controls, suggesting a potential up-regulation of α_1_-AR function that may contribute to the autonomic control in FGR lambs. This reliance on α_1_-AR function is supported by studies in carunclectomy-induced chronically hypoxic fetal sheep demonstrating a significantly greater hypotensive response to α_1_-AR blockade compared with normoxic foetuses at ~0.85 gestation [61]. Importantly, the maintenance of BP by α_1_-AR activity was not dependent on post-ganglionic sympathetic activation, signifying an organ-specific mechanism independent of neural stimuli to the adrenal medulla [62]. Despite this, contradicting evidence also exists regarding α_1_-AR modulation in the peripheral vasculature following chronic hypoxia, with sheep studies reporting either impaired [25,43] or enhanced [63,64] α_1_-adrenergic activity or abundance. Regardless, these data show that there is evolving dysfunction in α_1_-adrenergic function following chronic hypoxia, characterised as a greater reliance of central cardiovascular function (i.e. heart) to maintain cardiac output, with variable effects on the peripheral vasculature. We also found that non-asphyxiated FGR lambs had similar isolated cardiac responses to α_1_-AR activation compared with controls, suggesting that asphyxia independently modulates α-adrenergic activity in FGR hearts to maintain contraction under stress. Prolonged sympathetic activation can cause cardiomyocyte damage or apoptosis and worsens contractile function, thus increasing the risk of cardiovascular dysfunction in the long term [65].

We also interrogated the responses of the isolated heart to β_1_-AR stimulation, given its importance in maintaining fetal HR during brief asphyxic episodes in fetal sheep [38]. Similar to α_1_-adrenergic activation, asphyxiated FGR lambs had a significantly greater response to β_1_-adrenergic stimulation compared with asphyxiated controls. This response was only observed in the lowest dose of dobutamine, suggesting a maximal saturation of β_1_-ARs prevented dose-dependent increases in contractility. To date, studies in chronically hypoxic or FGR fetal sheep have only characterised impairments in cardiac function in response to a non-selective β-adrenergic agonist, isoproterenol [54,55,66]. We therefore chose to investigate the effects of a clinically relevant β_1_-AR agonist, dobutamine, given its common use in the neonatal intensive care unit to treat hypotensive preterm infants [3]. Activation of β_1_-ARs results in the activation of adenylyl cyclase and conversion of ATP into cAMP, ultimately phosphorylating ryanodine receptors and L-type Ca^2+^ channels to allow Ca^2+^ influx into the cytosol. β_1_-AR activation also phosphorylates SERCA/ATP2A2 to enhance calcium reuptake into the sarcoplasmic reticulum, overall resulting in positive inotropy via enhanced calcium cycling. Interestingly, the altered responses to both α_1_- and β_1_-adrenergic activation occurred independently of parenchymal or vascular adrenergic receptor abundance in the left ventricle and may therefore indicate a greater capacity for calcium handling in the cardiomyocyte. This potential for increased calcium handling is further supported by our finding of increased mRNA expression of SERCA/ATP2A2 in the left ventricles of FGR lambs. Similarly, a study by Lock et al. showed isolated cardiomyocytes from chronically hypoxic mice offspring raised into adulthood had a greater increase in systolic calcium amplitude after isoprenaline stimulation, suggesting sympathetic sensitisation [67]. Taken together, these data suggest that calcium handling may be developmentally programmed during gestation and persist beyond the transitional stage, potentially increasing the risk of sustained sympathetic activation and afterload and increasing the risk of long-term cardiac injury.

The findings of increased α_1_- and β_1_-adrenergic sensitivity underscore the need for tailored cardiovascular management strategies in FGR neonates, particularly during resuscitation when adrenergic activation may be altered. Optimising pharmacological support in FGR infants is critical, as standard dosing regimens may not account for the heightened adrenergic sensitivity observed in this population [6]. The precise interactions between adrenergic sensitivity, pharmacological interventions and cardiovascular outcomes in FGR neonates remain grossly understudied. Targeted research is required to determine whether tailored strategies can improve short- and long-term cardiovascular outcomes in this population. Potential strategies for personalised management that require further investigation include initiating resuscitation with lower doses of adrenaline, which may provide sufficient cardiovascular support while mitigating the risk of BP overshoot. In hypotensive FGR infants, α_1_-adrenergic agonists such as phenylephrine, oxymetazoline or methoxamine may offer more predictable haemodynamic control due to their direct myocardial and vasoconstrictive effects, while limiting β_1_-mediated variability. Where catecholamine therapy is inadequate or contraindicated, non-catecholamine agents such as vasopressin or milrinone may serve as alternative pathways for supporting vascular tone and cardiac output [68,69]. Notably, the absence of differences in the cardiac response to GTN suggests preserved NO-mediated vasodilation in FGR hearts, indicating that cardiovascular alterations in this cohort are primarily driven by changes in adrenergic, rather than NO-dependent mechanisms. In the present study, GTN was selected to assess NO-mediated responses due to its established clinical use and physiological relevance as an enzymatically activated NO donor that stimulates the NO-sGC-cGMP signalling pathway, in contrast to chemically decomposing donors, such as sodium nitroprusside [70,71].

There were strengths and limitations to this work. One key strength is our well-defined FGR model of asymmetrical growth restriction, indicated by an increased brain-to-body weight ratio with a preserved heart-to-body weight ratio, which recapitulates chronic hypoxia and acidemia following placental insufficiency [30]. Our lambs were also delivered preterm at a similar stage of cardiovascular development to a moderately preterm infant (32–34 weeks’ gestation) [31,32] to replicate the large proportion of FGR infants delivered moderately/late preterm to prevent stillbirth [72]. However, we also acknowledge that the investigation of a single gestational age is a limitation given that preterm birth comprises a broad spectrum of gestational ages and therefore morbidities. We also acknowledge the limitation that FGR foetuses likely received a higher per kilogram dose of antenatal betamethasone due to lower body weight. Although prior studies suggest that antenatal corticosteroids do not significantly alter neurodevelopmental [73] or cardiovascular [54] outcomes in FGR foetuses, future studies may adjust dosing for gestation-specific weight in FGR cohorts. Other limitations that may limit the clinical applicability of our findings include the lack of interrogation of intracellular calcium concentrations in isolated cardiomyocytes. We can therefore only extrapolate intracellular calcium handling based on the gene expression of the calcium transporter SERCA/ATP2A2. However, calcium handling also involves other transporters downstream of adrenergic receptor activation, including Protein Kinase A (PKA), Calcium/calmodulin-Dependent Protein Kinase II (CaMKII), Inositol 1,4,5-Trisphosphate (IP_3_) and ryanodine receptors [74,75], which may also provide further insight into the mechanisms underlying the changes in left ventricular contractility. We also acknowledge that our baseline blood gases highlight similar PaO_2_ levels between control and FGR groups, contrary to the characteristic hypoxia traditionally observed in SUAL-induced growth-restricted lambs [30]. This was likely due to the ventilation provided to the ewe during surgical instrumentation of the foetus, increasing placental oxygen transfer prior to collecting the arterial blood sample. Additionally, baseline haematocrit was significantly lower in FGR_ASPHYXIA_ lambs compared with FGR_VENT_ lambs. However, FiO_2_ requirements during ventilation did not differ significantly between FGR_VENT_ and FGR_ASPHYXIA_ lambs, indicating that oxygen delivery and gas exchange efficiency were not substantially compromised by the variation in haematocrit. This study also lacked an assessment of circulating catecholamine levels, which are known to maintain the chronic redistribution of cardiac output in chronically hypoxaemic fetal sheep and ultimately lead to a blunting of adrenergic receptor activity. However, previous studies have shown no differences in circulating epinephrine or norepinephrine levels in preterm 24-hour-old FGR lambs [43], nor were there differences in the catecholamine levels of asphyxiated preterm FGR and control lambs [27]. Therefore, it is unlikely that an increase in circulating catecholamine levels would have contributed to the altered response to asphyxia.

We have characterised the cardiovascular response between preterm FGR and control lambs to severe perinatal asphyxia by UCO. We have shown that FGR lambs experience delayed rebound hypertension after resuscitation due to poor myocardial and vascular contractility, which may lead to an inadequate perfusion of organs. Importantly, these changes likely reflect increased ventricular α_1_- and β_1_-AR function rather than altered receptor abundance, potentially involving up-regulation of downstream calcium handling effectors. Overall, these data show that FGR infants exposed to asphyxia may be prone to haemodynamic instabilities in the perinatal period, with alterations in sympathetic activation which may persist into the long term to potentially increase the risk of cardiovascular injury and cardiovascular disease later in life.

**Clinical Perspectives**

- Fetal growth restriction (FGR), often caused by placental insufficiency, leads to chronic fetal hypoxia that can alter the neonate’s cardiovascular responses to later hypoxic insults, such as perinatal asphyxia.
- In this study, FGR lambs displayed vascular dysfunction and abnormal cardiopulmonary haemodynamic responses to perinatal asphyxia, with consistently lower blood pressures during asphyxia, delayed rebound hypertension after resuscitation and heightened ventricular α₁- and β₁-adrenergic receptor responsiveness despite unchanged receptor abundance.
- These findings suggest that early life vascular and adrenergic dysregulation may predispose FGR infants to haemodynamic instability following exposure to additional insults, underscoring the need for tailored clinical management to reduce cardiovascular risk.

## Supplementary material

online supplementary material 1.

## Data Availability

Data will be made available by the authors upon reasonable request.

## Abbreviations

ANOVA: analysis of variance
ɑ1-AR: ɑ1-adrenergic receptor
AaDO_2_: alveolar-arterial difference in oxygen
BP: blood pressure
CPP: coronary perfusion pressure
CaMKII: Calcium/calmodulin-Dependent Protein Kinase II
DAB: 3,3’-Diaminobenzidine
FGR: fetal growth restriction
FiO_2_: fraction of inspired oxygen
GTN: glyceryl trinitrate
HR: heart rate
HRP: horseradish peroxidase
IP_3_: Inositol 1,4,5-Trisphosphate
IRP: isovolumetric relaxation period
LVDP: left ventricular developed pressure
MARP: Monash Animal Research Platform
NO: nitric oxide
NO: nitric oxide
NPPA: atrial natriuretic peptide
NPPB: B-type natriuretic peptide
PEEP: positive end-expiratory pressure
PIP: peak inspiratory pressure
PKA: Protein Kinase A
PaCO_2_: arterial partial pressure of carbon dioxide
PaO_2_: arterial partial pressure of oxygen
ROSC: return of spontaneous circulation
RT-qPCR: reverse transcription-quantitative polymerase chain reaction
SERCA/ATP2A2: sarcoplasmic/endoplasmic reticulum calcium ATPase 2
SUAL: single umbilical artery ligation
SaO_2_: saturation of oxygen
UCO: umbilical cord occlusion
VEI: ventilator efficiency index
cDNA: complementary DNA
crSO_2_: regional cerebral oxygenation
d: days
dGA: days gestation
dP/dt_max_: maximal rate of rise of left ventricular
dP/dt_min_: maximal rate of fall of left ventricular relaxation
β1-AR: β1-adrenergic receptor
β_1_-AR: β_1_-adrenergic receptor
