# Supplementary material for: In vivo and ex vivo cardiovascular haemodynamic responses of preterm growth-restricted lambs to perinatal asphyxia
Source: Clin Sci (Lond). 2025 Dec 23;139(24):1709–32. doi: 10.1042/CS20258191 (PMC12794318; doi:10.1042/CS20258191)
Supplement: online supplementary material 1. [file cs-139-24-CS20258191-s001.docx]

**Supplementary Data**

**In Vivo and Ex Vivo Cardiovascular Haemodynamic Responses of Preterm Growth-Restricted Lambs to Perinatal Asphyxia**

Zahrah Azman^1,2^, Beth R. Piscopo^1,2^, Amy E. Sutherland^1^, Alison Thiel^1^, Valerie A. Zahra^1^, Yen Pham^1^, Ilias Nitsos^1^, Mumu Mahjabin Hossain^1,2^, Atul Malhotra^1,3,4^, Suzanne L. Miller^1,2^, Kristen J. Bubb^5,6^, Graeme R. Polglase^1,4^* and Beth J. Allison^1,2^*

^1^ The Ritchie Centre, Hudson Institute of Medical Research, Clayton, VIC, Australia

^2^ Department of Obstetrics and Gynaecology, Monash University, Clayton, VIC, Australia

^3^ Monash Newborn, Monash Medical Centre, Clayton, VIC, Australia

^4^ Department of Paediatrics, Monash University, Clayton, VIC, Australia

^5^ Biomedicine Discovery Institute, Monash University, Clayton, VIC, Australia

^6^ Victorian Heart Institute, Monash University, Clayton, VIC, Australia

* These authors contributed equally.

**Corresponding author:**

Zahrah Azman

[siti.azman@monash.edu](mailto:beth.allison@hudson.org.au)

27-31 Wright St

Clayton, VIC 3168

Australia

**Co-corresponding author:**

Dr Beth Allison

[beth.allison@hudson.org.au](mailto:beth.allison@hudson.org.au)

27-31 Wright St

Clayton, VIC 3168

Australia

**Supplementary Figures**

**
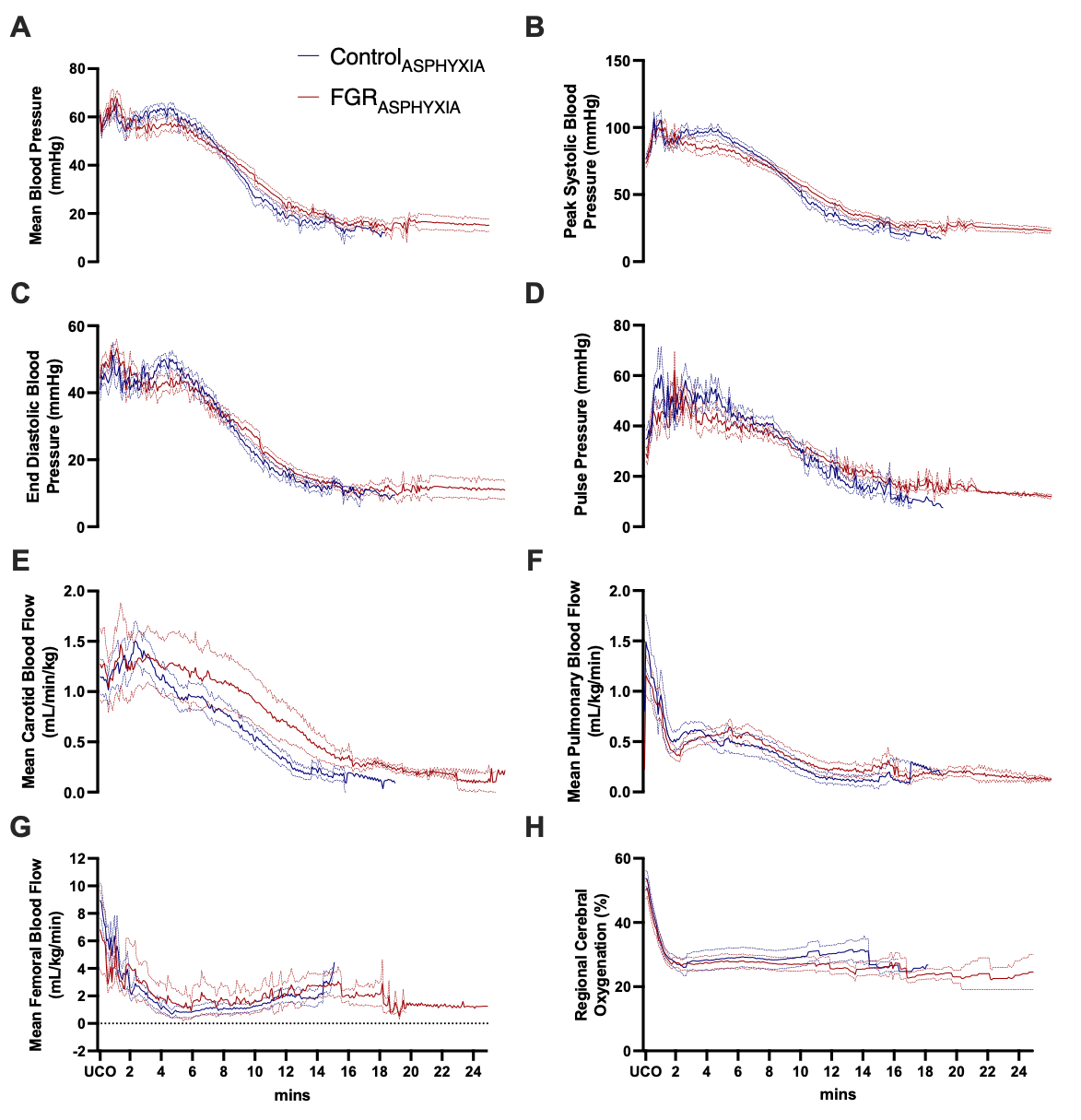
**

***Supplementary Figure S1. Real-time physiological responses to asphyxia.*** Data presented as mean ± standard error of the mean of (**A**) mean arterial blood pressure, (**B**) peak systolic blood pressure, (**C**) end diastolic blood pressure, (**D**) pulse pressure, (**E**) mean carotid blood flow (corrected for brain weight), (**F**) mean pulmonary blood flow (corrected for lung weight), (**G**) mean femoral blood flow (corrected for body weight) and (**H**) mean regional cerebral oxygenation. Groups are asphyxiated control (Control_ASPHYXIA_, n=12) and asphyxiated FGR (FGR_ASPHYXIA_, n=11) lambs. Data analysed via repeated measure mixed effects analysis. As real-time data are influenced by varying attrition rates, the physiological responses to asphyxia were instead presented as quartiles relative to each lamb’s individual asphyxia duration in the main manuscript.


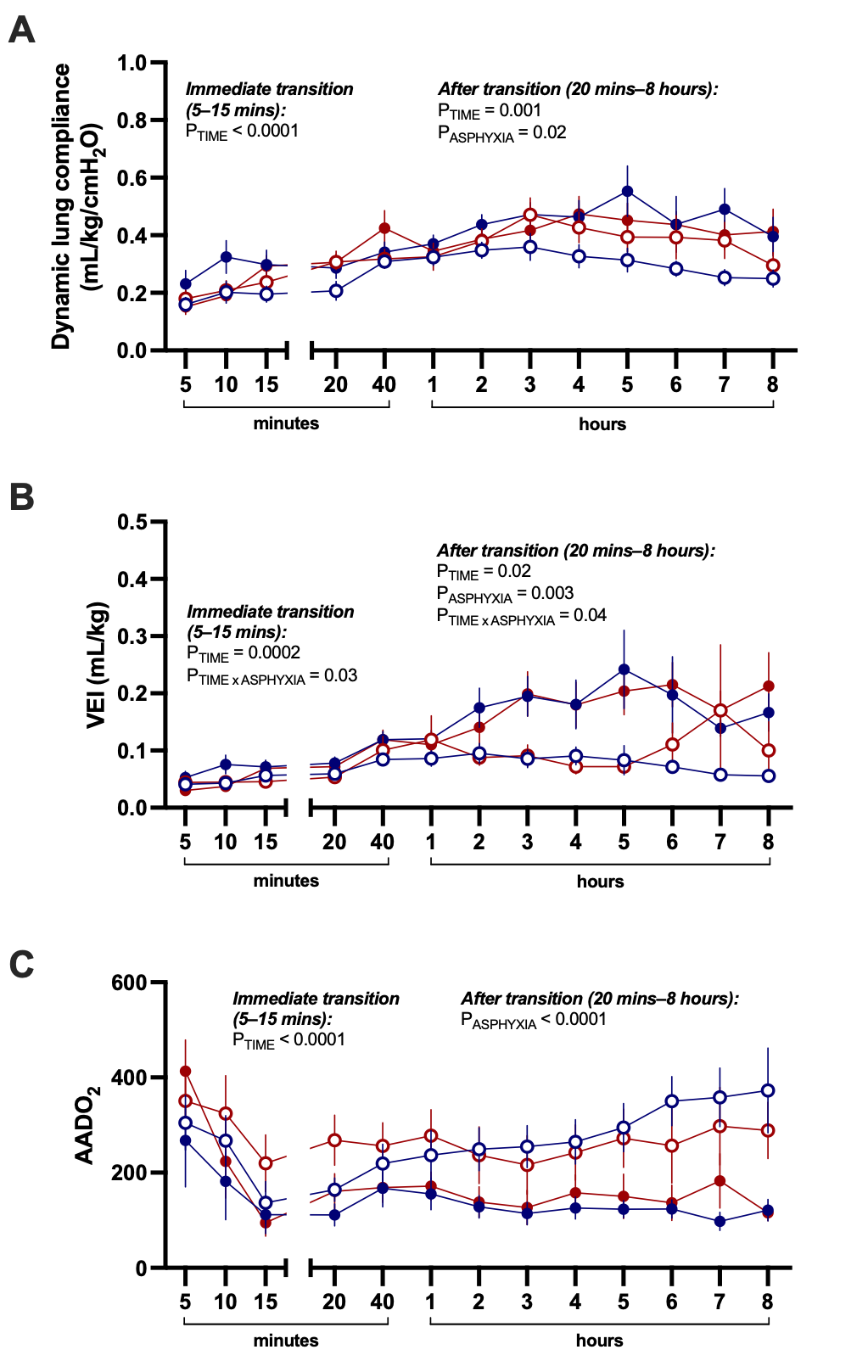


***Supplementary Figure S2. Ventilatory parameters.*** Data presented as mean ± standard error of the mean of (**A**) dynamic lung compliance, (**B**) ventilation efficiency index (VEI), and (**C**) alveolar-arterial oxygen difference (AaDO_2_). Groups are asphyxiated control (Control_ASPHYXIA_, n=12) and asphyxiated FGR (FGR_ASPHYXIA_, n=11) lambs. Statistical analyses were performed using repeated measure mixed effects analysis, conducted separately for the immediate transition period (5–15 min) and the post-transition period (20 min–8 h).


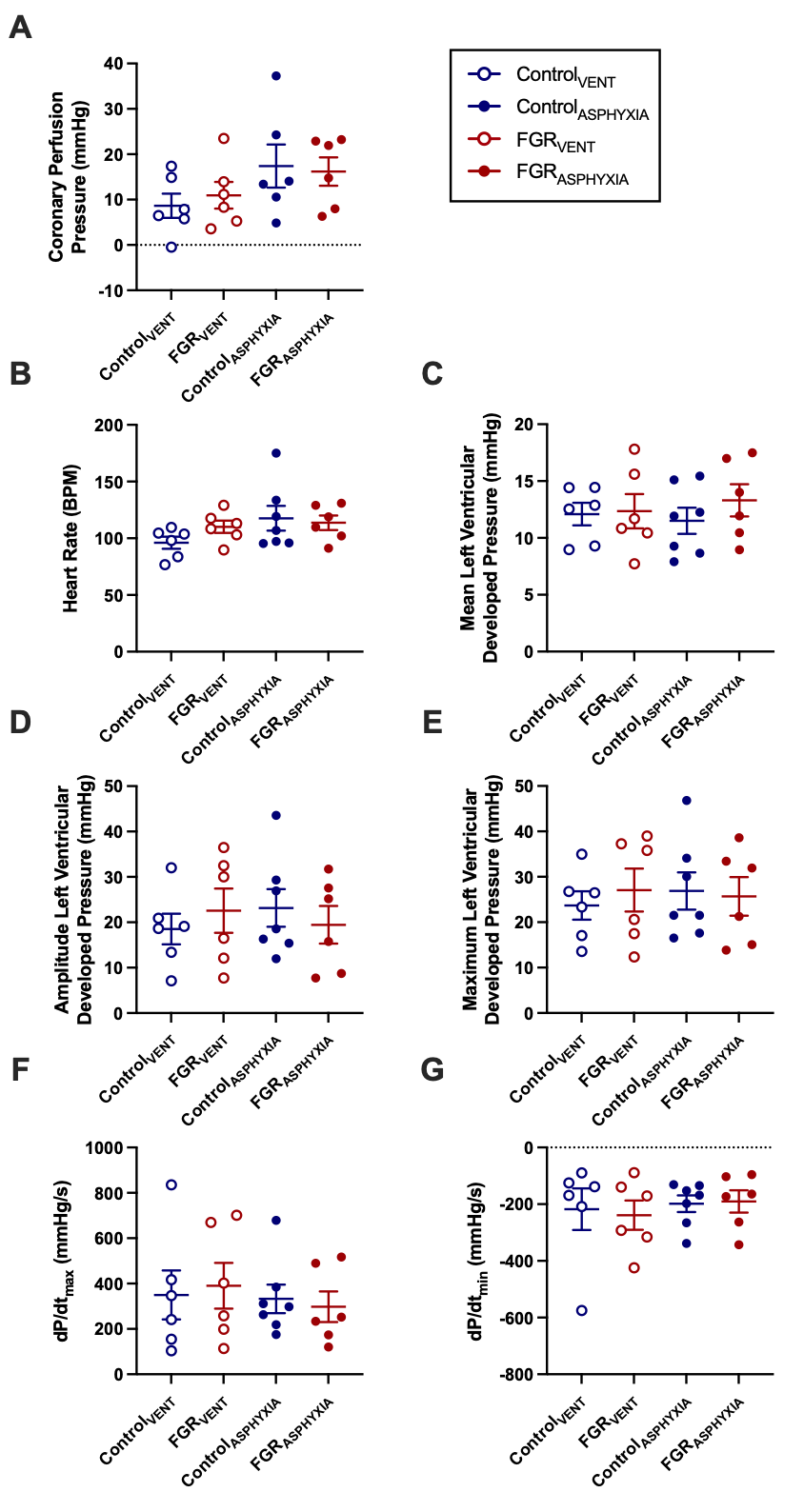


***Supplementary Figure S3. Baseline ex vivo cardiac function.*** Data presented as mean ± standard error of the mean of (**A**) coronary perfusion pressure, (**B**) heart rate, (**C**) mean left ventricular developed pressure, (**D**) amplitude of left ventricular developed pressure, (**E**) maximum left ventricular developed pressure, (**F**) maximal left ventricular contractility (dP/dt_max_) and (**G**) minimal left ventricular contractility (dP/dt_min_). Groups are ventilated control (Control_VENT_, n=6), ventilated FGR (FGR_VENT_, n=6), asphyxiated control (Control_ASPHYXIA_, n=7), and asphyxiated FGR (FGR_ASPHYXIA_, n=6) lambs. Data analysed via mixed effects analysis; statistical significance threshold *p*<0.05.


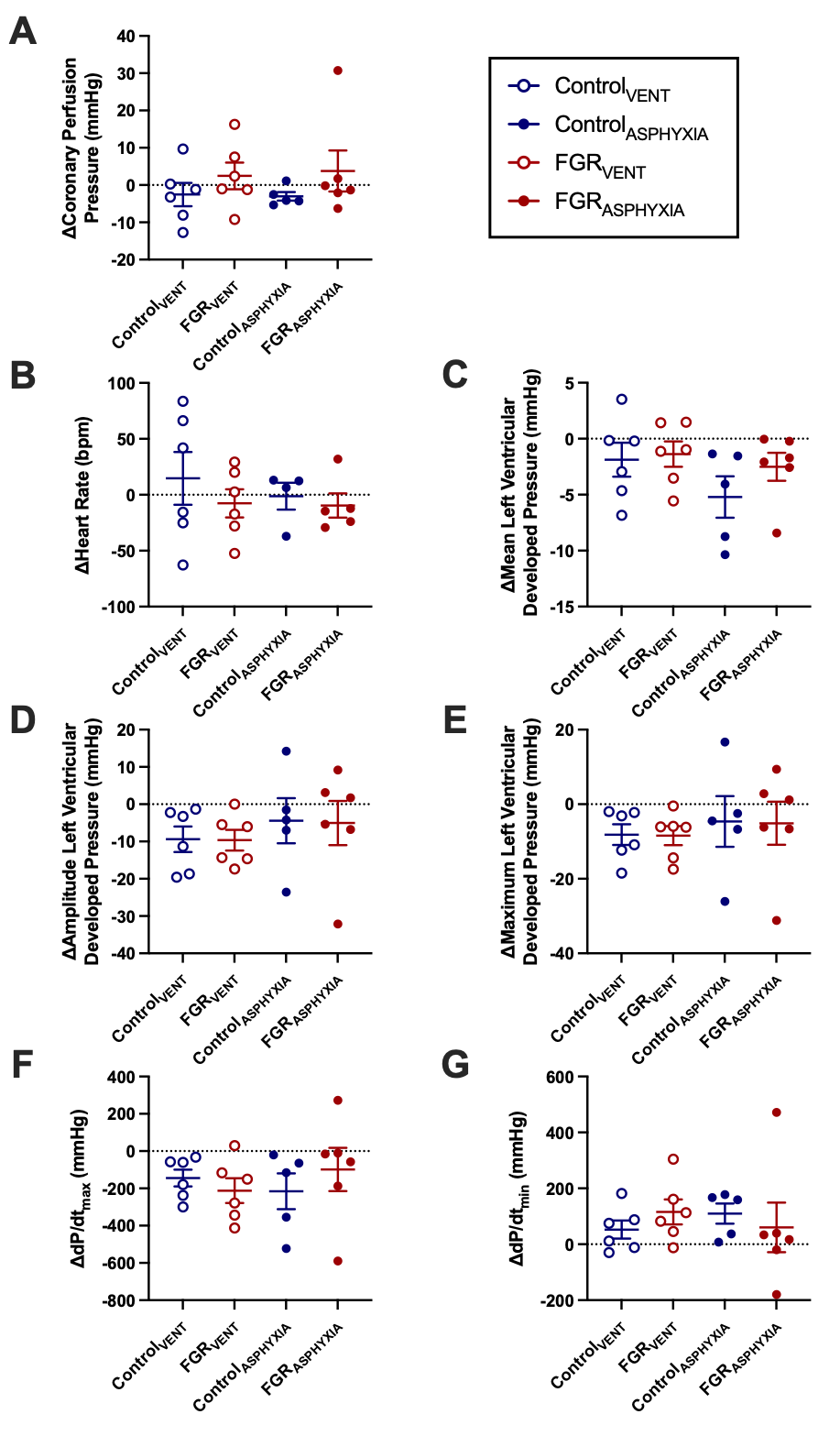


***Suppelementary Figure S4. Ex vivo cardiac responses to glyceryl trinitrate.*** Data presented as mean ± standard error of the change in (**A**) coronary perfusion pressure, (**B**) heart rate, (**C**) mean left ventricular developed pressure, (**D**) amplitude of left ventricular developed pressure, (**E**) maximum left ventricular developed pressure, (**F**) maximal left ventricular contractility (dP/dt_max_) and (**G**) minimal left ventricular contractility (dP/dt_min_) in response to a bolus dose of glyceryl trinitrate. Groups are ventilated control (Control_VENT_, n=6), ventilated FGR (FGR_VENT_, n=6), asphyxiated control (Control_ASPHYXIA_, n=5), and asphyxiated FGR (FGR_ASPHYXIA_, n=6) lambs. Data analysed via mixed effects analysis.


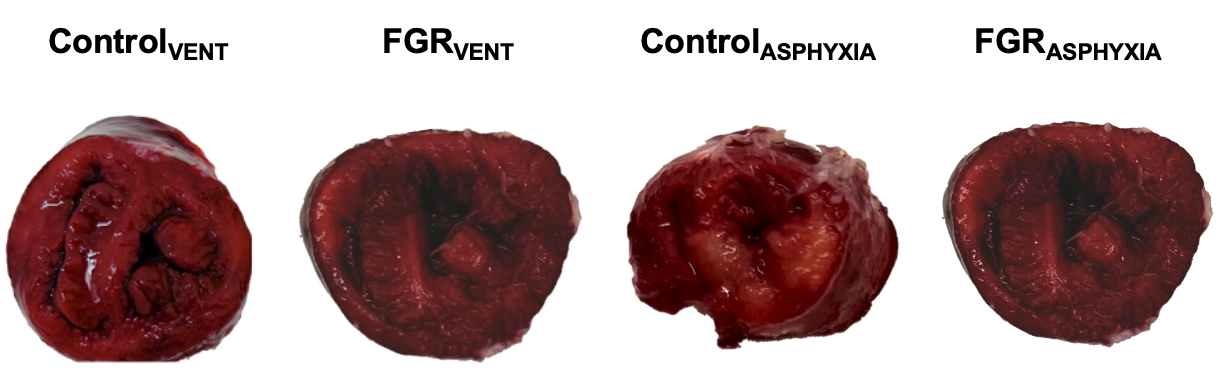


***Supplementary Figure S5. Representative images of infarct sizes.*** Representative images of ventricles incubated in 1% 2,3,5-triphenyltetrahydrozolium chloride to visualise infarct size. Viable tissue is stained red by interaction with lactate dehydrogenase, while areas of infarct remain unstained or white. Groups are ventilated control (Control_VENT_, n=6), ventilated FGR (FGR_VENT_, n=6), asphyxiated control (Control_ASPHYXIA_, n=5), and asphyxiated FGR (FGR_ASPHYXIA_, n=6) lambs.**Supplementary Tables**

***Supplementary Table S1. Postmortem cardiac dimensions***

|  | **Control_VENT_** | **FGR_VENT_** | **Control_ASPHYXIA_** | **FGR_ASPHYXIA_** | **p-value** |
| --- | --- | --- | --- | --- | --- |
| **Globularity (AU)** | 0.58 ± 0.01 | 0.56 ± 0.01* | 0.59 ± 0.01 | 0.54 ± 0.01* | **P_FGR_ = 0.014**  P_ASPHYXIA_= 0.75  P_INT_ = 0.20 |
| **Heart length (mm)** | 6.6 ± 0.22 | 6.9 ± 0.15 | 6.7 ± 0.1 | 6.4 ± 0.1 | P_FGR_ = 0.93  P_ASPHYXIA_= 0.32  P_INT_ = 0.09 |
| **Heart width (mm)** | 4.4 ± 0.20 | 4.3 ± 0.12 | 4.4 ± 0.10 | 4.0 ± 0.09 | P_FGR_ = 0.11  P_ASPHYXIA_= 0.18  P_INT_ = 0.23 |

Data expressed as mean ± standard error of the mean and analysed by 2-way ANOVA. Statistical significance threshold **p* < 0.05 FGR vs. control. *AU: arbitrary units; FGR: fetal growth restriction.*
